# The global health and economic impact of low-back pain attributable to occupational ergonomic factors in the working-age population by age, sex, geography in 2019

**DOI:** 10.5271/sjweh.4116

**Published:** 2023-10-01

**Authors:** Ningjing Chen, Daniel Yee Tak Fong, Janet Yuen Ha Wong

**Affiliations:** 1 School of Nursing, Putian University, Putian, China.; 2 School of Nursing, Li Ka Shing Faculty of Medicine, The University of Hong Kong, Hong Kong, China.; 3 School of Nursing and Health Studies, Hong Kong Metropolitan University, Hong Kong, China.

**Keywords:** healthcare cost, productivity loss, years lived with disability

## Abstract

**Objective:**

Occupational ergonomic factors (OEF) include physical exertion, demanding posture, repetitive work, hand-arm vibration, kneeling or squatting, rising, and climbing, which are risk factors for low-back pain (LBP). This study aimed to examine the prevalence, years lived with disability (YLD), healthcare costs, and productivity losses of LBP attributable to OEF by age, sex, World Health Organization region, and country in 2019.

**Methods:**

In this cross-sectional study, prevalence and YLD were extracted from the Global Burden of Diseases, Injuries, and Risk Factors Study 2019. Employment statistics were obtained from the International Labour Organization websites. Health and economic impact was estimated for 192 countries and territories using the population attributable fraction method.

**Results:**

Globally, OEF were responsible for 126.1 million prevalent cases of LBP and 15.1 million YLD in the working-age population (aged 15–84 years) in 2019, with the Western Pacific region suffering most. OEF-attributable LBP led to $216.1 billion of economic losses worldwide. Of these, $47.0 billion were paid in healthcare costs, with the public sector serving as the largest contributor (59.2%). High-income countries bore >70% of global economic burden, whereas middle-income countries experienced >70% of global YLD. Generally, more prevalent cases and healthcare costs were found among females, whereas more YLD, productivity losses, and total costs were found among males.

**Conclusions:**

Globally, OEF-attributable LBP presented a heavy burden on health and economic systems. Exercise together with education, active monitoring, evidence-based medical practices, alternative cost-effective solutions, and prioritizing health policies are needed.

Low-back pain (LBP) refers to pain on the posterior area between the lower 12^th^ rib margins to the lower gluteal folds that continues for at least one day, which may not co-exist with lower limb pain (1). In 2019, the global number of prevalent cases of LBP increased to 568.4 million, contributing to 63.7 million years lived with disability (YLD) (2). Thus, LBP became the leading driver of global YLD in 2019 (2).

The large amount of morbidity posed a great challenge to the global health and economic systems. In 2019, 568.4 million people with LBP worldwide were in need of rehabilitation, and unmet rehabilitation services existed in both developing and developed countries (3). Additionally, from 1996 to 2016, healthcare spending on low-back and neck pain in the US grew at 5.3% annually, which increased to US$134.5 billion and ranked 1^st^ among 154 health conditions in 2016 (4). In addition to the large economic burden stemming from healthcare expenditures, LBP also contributed to great losses in productivity. Presenteeism due to chronic back pain led to US$1920 productivity losses per head in the US annually (5). In less developed countries (eg, Brazil), LBP cost the Brazilian economic system US$500 million annually from 2012 to 2016, with 79% of costs in productivity losses (6).

Occupational ergonomic factors (OEF) include physical exertion, demanding posture, repetitive work, hand-arm vibration, kneeling or squatting, rising, and climbing (7). Exposure to OEF increases the risk for LBP (8). As estimated in the Global Burden of Diseases, Injuries, and Risk Factors Study (GBD), OEF accounted for 8.1 and 7.3 million YLD in males and females, respectively (9). Although the previous Lancet study (8) has provided data on YLD attributable to risk factors for 369 diseases and injuries, other disease burden estimates (eg, prevalence) attributable to risk factors have not been quantified. Currently, only few countries have estimated the national economic burden of LBP (4, 10–13). In addition, it is difficult to compare the economic estimates of LBP directly across countries due to differences in currencies. Moreover, disease-related costs not only included direct costs (ie, healthcare spending) but also indirect costs (ie, productivity losses) (14). However, far fewer studies have investigated the productivity losses caused by LBP (11, 12, 15). Additionally, the existing literature has not investigated how the global economic burden of LBP is attributable to OEF. Furthermore, in most countries, the public (eg, funding from government budgets), private (eg, financial resources from private companies or insurance), and out-of-pocket sectors serve as the main financial providers. However, most of the current economic analyses of LBP (10–12, 15) only provide an overall picture of how large the economic burden that a country bore, not investigating further where the financial support was derived.

Therefore, this study aimed to quantify the global prevalence, morbidity, healthcare costs, and productivity losses of OEF-attributable LBP. We also evaluated how the economic burden was distributed among the public, private, and out-of-pocket sectors, which might provide insight into making concerted efforts to launch joint initiatives through cross-sector collaboration.

## Methods

### Data sources

The GBD 2019 study provides the most comprehensive and systematic estimates on epidemiology levels and changing patterns for 369 diseases and injuries, and 87 risk factors (1). We extracted prevalence estimates, OEF-attributable YLD, and population attributable fraction (PAF) for the age-standardized YLD rate of LBP in 2019 from the GBD data portal (16). In the GBD program, the 2.5^th^ and 97.5^th^ percentiles of an estimate were derived from the 1000 ranked draws, which constructed its 95% uncertainty intervals (UI). In addition, we also obtained the labor income share (17), and workforce participation rate in each age group (18) from the International Labour Organization (ILO) websites. Statistics on labor force were extracted from the World Bank and the ILO data repositories (19, 20). Additionally, gross domestic product (GDP) estimates were extracted from the World Health Organization (WHO) (21), and the World Bank datasets (19). The shares that three sectors contribute to healthcare costs were extracted from the WHO data portal (21). Details are described in Methodological Appendix and supplementary material (www.sjweh.fi/article/4116) S1–S4.

### Exposure

Because the occupational exposure level associated with LBP by job category has not been quantified adequately in each country or territory, occupation served as a proxy for OEF in the GBD project (22, 23). Although this might introduce bias, the potential interaction among various exposures was not necessarily to be considered because each occupation reflects the combined effects of various exposures concerned, including those from physical and psychosocial stressors (22). Therefore, OEF are defined as the proportion of the working population exposed to work that leads to LBP, on the basis of population distributions in seven occupations (8, 22). These occupational categories include professional, technical and related workers; administrative and managerial workers; clerical and related workers; sales workers; service workers; agriculture, animal husbandry and forestry workers, fishermen and hunters; and production and related workers, transport equipment operators and laborers (8, 22).

The GBD team classified the occupations into these categories based on the economic characteristics, similar exposures at the physical and psychosocial levels as well as the evidence from previous research (22). The theoretical minimum-risk exposure is the exposure level of clerical and related workers (8, 22), who are considered to have no-to-little exposure to occupational factors (22). Therefore, the relative risk in clerical and related workers is 1. The relative risks for LBP by age and occupation group in the GBD 2019 study (8) are shown in supplementary table S5.

### Statistical analysis

We estimated the prevalent cases, YLD, healthcare costs, and productivity losses of LBP attributable to OEF by sex and age using the PAF approach. Healthcare costs per case were estimated using an extrapolation method. Productivity losses were estimated with the labor income per worker. The UI of our estimates were obtained from a sensitivity analysis. Details on estimation are described in the subsections below and Methodological Appendix. To ensure the comparability of economic impact across countries and territories, all costs were reported in US dollars (US$). Data analyses were conducted in RStudio Version 1.3.1093.

### Estimation of prevalent cases and YLD

The PAF describes the proportion of risk that would be reduced if the exposure to a risk factor was limited to the theoretical minimum risk level (8). Details on the estimation of PAF are provided in Methodological Appendix. We calculated the number of prevalent cases of LBP attributable to OEF in each age and sex group by multiplying the number of prevalent cases of LBP in each age and sex group by the PAF for the age-standardized YLD rate. The number of YLD counts of LBP attributable to OEF was extracted from the GBD result tool (16). The attributable prevalent cases and YLD were summed across ages and sexes to generate the total attributable prevalent cases and YLD.

### Estimation of healthcare costs

So far, no datasets have reported the healthcare costs of LBP in each country. For this reason, we aimed to calculate the healthcare costs of LBP in each country by extrapolating a representative baseline estimate with a country-specific cost weight. Consistent with a previous study (24), we performed a comprehensive search of current studies estimating national healthcare costs of LBP, which were evaluated and ranked based on serval criteria (Methodological Appendix, supplementary figure S1 and table S6). Finally, we used the economic estimates in the US (4) to provide baseline data and calculated healthcare costs in each country and territory with the method described below.

We first calculated healthcare costs of LBP in the US (supplementary table S7). Subsequently, healthcare spending per head in each country was extracted from a previous Lancet study (25). A country-specific cost weight was equal to healthcare spending per head in the target country divided by that in the US. We arrived at healthcare costs per case of LBP in each country by multiplying the country-specific cost weight by healthcare costs per case in the US. Generally, healthcare costs per case estimated using this method were comparable to previous estimates (supplementary figure S2) (10–13). Lastly, we multiplied healthcare costs per case by the number of OEF-attributable prevalent cases of LBP. This quantity was summed across ages and sexes to arrive at healthcare costs of OEF-attributable LBP in each country. Details on the estimation are in Methodological Appendix and supplementary table S4. We also estimated the healthcare costs borne by the public, private, and out-of-pocket sectors by applying the spending shares (21), which was based on the assumption that the distributions of healthcare costs among the three sectors at the national level applied to that of LBP.

### Estimation of productivity losses

Generally, productivity includes market and non-market productivity. In this cross-sectional study, we estimated market productivity based on the labor income per worker, which was equal to the labor income share multiplied by GDP and divided by the size of the labor force. In each age group, given that not all people participated in the workforce, we adjusted the labor income per worker with the labor force participation rate. In this study, we estimated the disease and economic impact of OEF-attributable LBP in the working-age (ie, ≥15 years) population. We assumed the labor force participation rate to be zero in people aged ≥85 years. Therefore, YLD and productivity losses were estimated among workers aged 15–84 years.

OEF-associated morbidity also results in reduced non-market production, which is often estimated separately from GDP. Based on previous estimates, non-market production was equal to 23% (26) of the US’ GDP and 35% (27) of Ghana’s GDP. As most countries did not release non-market production estimates, we assumed that non-market production equally contributed to 23% of GDP in high-income and upper-middle-income countries, and 35% in low-income and lower-middle-income countries. Subsequently, the productivity losses of OEF-attributable LBP, by sex and age, were estimated by multiplying the sum of the market and non-market output per worker by the number of YLD counts, which were summed across ages and sexes to generate the OEF-attributable productivity losses. This approach for calculating productivity losses had been applied in previous studies (27, 28). Additional information on estimation methods is in Methodological Appendix.

### Sensitivity analysis

We evaluated the lower and upper limits of our estimates (ie, UI) by repeating the analysis with the lower and upper bounds of all input variables. For those input variables without UI (eg, GDP), the baseline values of the input variables were used to calculate the minimum, mean, and maximum estimates (supplementary table S4).

## Results

In this study, we included 192 countries and territories with available data. Globally, OEF accounted for 126.1 million prevalent cases and 15.1 million YLD of LBP. The largest disease burden was found in the Western Pacific region with 34.4 million prevalent cases and 4.2 million YLD. In comparison, the smallest burden was observed in the Eastern Mediterranean region with 8.4 million prevalent cases and 1.1 million YLD (supplementary table S8).

In 2019, summed across ages and sexes, the healthcare costs of LBP attributable to OEF were $47.0 billion globally. By WHO region, $22.1 billion healthcare costs were spent in the Americas, $12.0 billion in Europe, $9.4 billion in the Western Pacific, $1.8 billion in Southeast Asia, $1.2 billion in the Eastern Mediterranean, and $0.6 billion in Africa (table 1). Healthcare costs by country are in supplementary table S9. Globally, OEF-associated healthcare costs were responsible for 21.7% of total costs. Specifically, this percentage was 25.2% in the Americas, 23.1% in the Eastern Mediterranean, 20.0% in Europe, 19.0% in the Western Pacific, 17.0% in Southeast Asia, and 14.8% in Africa (table 1). The percentage that healthcare costs accounted for total costs by country is in supplementary table S10.

**Table 1:** Healthcare costs, morbidity-related, and total costs of low-back pain attributable to occupational ergonomic factors by World Health Organization region in 2019. [UI=uncertainty interval.]

	Healthcare costs			Morbidity-related costs			Total costs
	US$ millions (UI)	% ^a^ of total		US$ millions (UI)	% ^a^ of total		US$ millions (UI)
Global	46 982 (32 327–66 463)	21.7		169 154 (100 910–261 825)	78.3		216 136 (133 237–328 287)
Africa	581 (371–873)	14.8		3347 (1909–5357)	85.2		3928 (2281–6230)
The Americas	22 082 (16 183–29 556)	25.2		65 487 (40 653–97 997)	74.8		87 569 (56 836–127 554)
Eastern Mediterranean	1230 (782–1862)	23.1		4097 (2338–6557)	76.9		5327 (3120–8419)
Europe	11 950 (7831–17 573)	20.0		47 761 (27 963–74 841)	80.0		59 711 (35 794–92 414)
Southeast Asia	1772 (1141–2658)	17.0		8633 (4984–13 758)	83.0		10 405 (6125–16 416)
Western Pacific	9367 (6019–13 940)	19.0		39 828 (23 063–63 314)	81.0		49 195 (29 081–77 254)

^a^ The percentage calculations were based on the mean values.

Generally, the contribution to global healthcare costs varied greatly across regions and sectors. Worldwide, the public sector was the largest contributor to global healthcare costs, bearing 59.2% of healthcare costs, ranging from 72.0% in Europe to 36.2% in Africa. In contrast, the out-of-pocket sector contributed least (19.4%, $9.1 billion) to global healthcare costs. However, household payments accounted for >40% (41.7%, $0.7 billion) of healthcare costs in Southeast Asia. Another 21.4% ($10.1 billion) of global healthcare costs were derived from private resources, which accounted for more than one-third (35.1%, $7.8 billion) of healthcare costs in the Americas (table 2). Health care costs by sector and country are in supplementary table S11.

**Table 2:** Healthcare costs of low-back pain attributable to occupational ergonomic factors borne by sector and World Health Organization region in 2019. [UI=uncertainty interval.]

	Public sector			Private sector/third party			Out-of-pocket sector	
	US$ millions (UI)	% ^a^		US$ millions (UI)	% ^a^		US$ millions (UI)	% ^a^
Global	27 798 (19 005–39 516)	59.2		10 062 (7225–13 721)	21.4		9105 (6083–13 202)	19.4
Africa	211 (134–318)	36.2		156 (100–234)	26.9		214 (137–321)	36.9
The Americas	11 370 (8323–15 236)	51.5		7750 (5730–10 288)	35.1		2942 (2116–4006)	13.3
Eastern Mediterranean	634 (404–956)	51.6		164 (105–248)	13.4		431 (273–658)	35.1
Europe	8599 (5640–12 634)	72.0		1031 (676–1514)	8.6		2322 (1516–3427)	19.4
Southeast Asia	776 (500–1161)	43.8		258 (166–386)	14.5		739 (475–1111)	41.7
Western Pacific	6208 (4003–9210)	66.3		702 (448–1050)	7.5		2456 (1566–3679)	26.2

^a^ The percentage calculations were based on the mean values.

**Figure 1:**
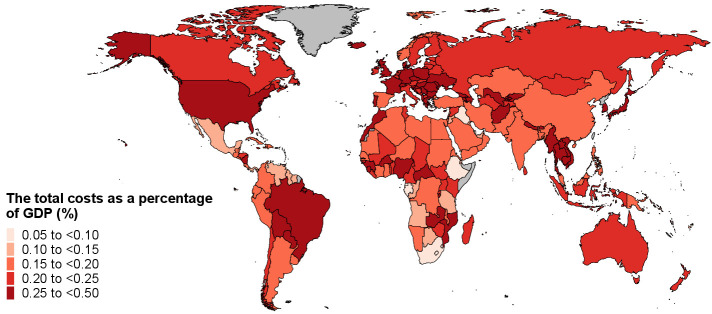
Total costs as a proportion of gross domestic product (GDP), 2019. Note: Grey areas represent countries or territories with no available data.

In 2019, OEF cost the global economy $169.2 billion in productivity losses. Of these, $65.5 billion were in the Americas, $47.8 billion in Europe, $39.8 billion in the Western Pacific, $8.6 billion in Southeast Asia, $4.1 billion in the Eastern Mediterranean, and $3.3 billion in Africa (table 1). In 2019, taking both healthcare costs and productivity losses into account, OEF were responsible for a total cost of $216.1 billion around the world. Globally, the total economic costs represented 0.25% of GDP, ranging from 0.06% in Gabon to 0.49% in Serbia (figure 1).

Table 3 presents the global healthcare costs, productivity losses due to morbidity, and total economic losses of OEF by WHO region and World Bank income level. Generally, the economic costs were not in direct proportion to population estimates and morbidity. For example, 13.2% of global population and 14.4% of global YLD were found in the Americas, wherein nearly half (47.0%) of global healthcare costs and 38.7% of global productivity losses were also observed. In contrast, Southeast Asia had approximately one-fourth (25.9%) of global population with 23.7% of global YLD, accounting for 3.8% of global healthcare costs and 5.1% of global productivity losses. Particularly, although greater than 70% of the economic burden (74.8% of global healthcare costs and 71.3% of global productivity losses) were found in high-income countries, middle-income countries bore more than 70% (73.3%) of global YLD.

**Table 3:** Healthcare costs, productivity losses due to morbidity, and years lived with disability (YLD) of low-back pain attributable to occupational ergonomic factors by World Health Organization (WHO) region and World Bank income group, 2019.

		Population (millions)		Healthcare costs (millions)		Per capita health care costs		Morbidity-related costs (millions)		Per capita morbidity costs		YLD (thousands)		YLD per 1000 persons
		N (%)		US$ (%)		US$		US$ (%)		US$		N (%)		N
Global		7628 (100.0)		46 982 (100.0)		6.2		169 154 (100.0)		22.2		15 128 (100.0)		2.0
WHO region														
	Africa	1092 (14.3)		581 (1.2)		0.5		3347 (2.0)		3.1		1999 (13.2)		1.8
	The Americas	1010 (13.2)		22 082 (47.0)		21.9		65 487 (38.7)		64.8		2176 (14.4)		2.2
	Eastern Mediterranean	697 (9.1)		1230 (2.6)		1.8		4097 (2.4)		5.9		1080 (7.1)		1.5
	Europe	930 (12.2)		11 950 (25.4)		12.8		47 761 (28.2)		51.3		2125 (14.0)		2.3
	Southeast Asia	1976 (25.9)		1772 (3.8)		0.9		8633 (5.1)		4.4		3586 (23.7)		1.8
	Western Pacific	1923 (25.2)		9367 (19.9)		4.9		39 828 (23.5)		20.7		4162 (27.5)		2.2
Income group														
	High	1197 (15.7)		35 161 (74.8)		29.4		120 673 (71.3)		100.8		2935 (19.4)		2.5
	Upper-middle	2895 (37.9)		9297 (19.8)		3.2		37 189 (22.0)		12.8		6097 (40.3)		2.1
	Lower-middle	2909 (38.1)		2309 (4.9)		0.8		10 635 (6.3)		3.7		4995 (33.0)		1.7
	Low	627 (8.2)		214 (0.5)		0.3		657 (0.4)		1.0		1100 (7.3)		1.8

**Figure 2:**
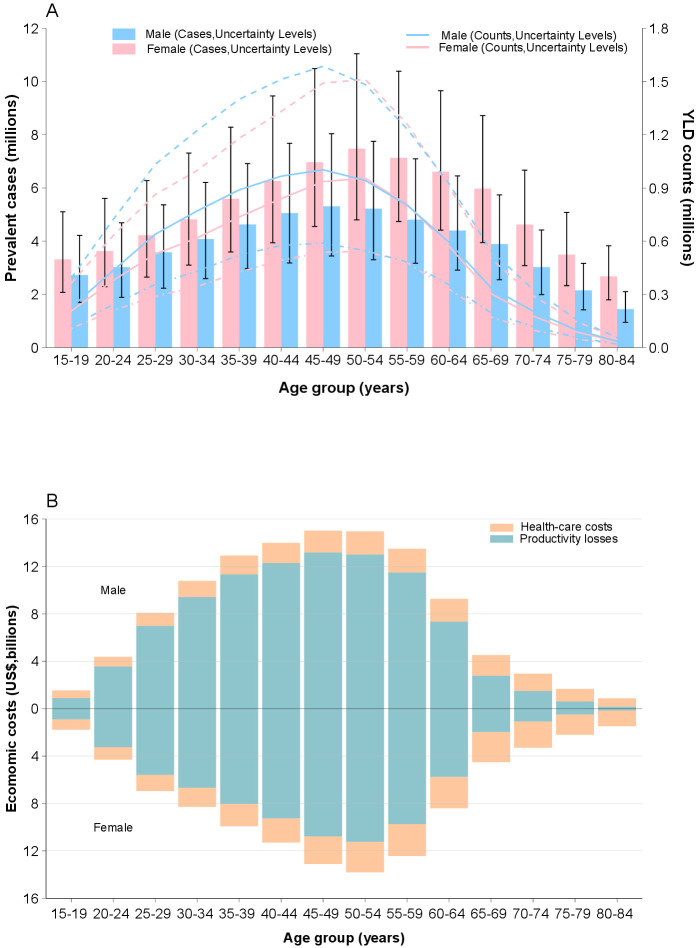
Health and economic burden of low -back pain attributable to occupational ergonomic factors by age and sex, 2019.

The number of prevalent cases of LBP attributable to OEF was larger in females across all ages and increased with age. In addition, the prevalence estimates peaked at the 45–49 and 50–54 years age group in males and females, respectively. Decreasing trends presented after the peak points. On the contrary, the number of YLD was generally larger among males across all ages except for the 50–59 years age group. The association between age and YLD estimates resembled those between age and prevalence estimates (figure 2, panel A).

Healthcare costs were higher among females across ages, whereas productivity losses were higher among males across ages except for the 15–19 years age group. Overall, the total costs were higher among males except for the 15–19 and 70–84 years age groups. Healthcare costs and productivity losses increased to the peak points at the 55–59 and 45–49 years age groups, respectively, and then turned to decreasing trends until the oldest group among males. The age patterns of the economic burden among females resembled those of males (figure 2, panel B).

In the sensitivity analysis, with the use of the UI of all input variables, OEF were responsible for 81.6–187.8 million prevalent cases, 8.7–24.1 million YLD, $32.3–66.5 billion in healthcare costs, $100.9–261.8 billion in productivity losses, and $133.2–328.3 billion in total economic losses due to OEF-attributable LBP in 2019 globally (table 1, and supplementary tables S8–S10).

## Discussion

This was the first study to evaluate the global health and economic burden of LBP attributable to OEF. Using a robust methodology, we estimated that 126.1 million cases of LBP with 15.1 million YLD were attributable to OEF in 2019 globally. We estimated the economic losses of OEF-attributable LBP to be $216.1 billion worldwide, with $47.0 billion in healthcare costs. In addition, the public sector was the largest contributor (59.2%) to global healthcare costs, followed by the private (21.4%) and out-of-pocket sectors (19.4%).

Generally, the disease burden did not align with the economic burden. High-income countries suffered a larger economic impact, whereas middle-income countries bore a higher disease burden. The disease burden of LBP was expected to increase in the following decades, especially in low-income and middle-income countries (29). For example, a previous study projected that the number of Chinese people with LBP in need of rehabilitation would increase to 465.9 million in 2030 (30). However, in sharp contrast to high-income countries, healthcare systems in low-income and middle-income countries tended to be fragile and not well-equipped to tackle the substantial burden (29). Therefore, job improvement and better occupational prevention were urgently needed. Previous efforts have been made to reduce the burden of LBP by mitigating the impact of OEF, such as launching physical exercise programs, providing education on lifting and working techniques, and encouraging the use of back support belts (31). For example, a hamstring stretch and spine exercise program was implemented in 136 workers from the manufacturing industry, with 25% of participants in the intervention group reporting pain relief after a three-month follow-up. In contrast, this percentage was only 12% in the control group without exercise (32). A previous review also shows moderate quality evidence that exercise together with education contributes to a future reduction of LBP episodes with a relative risk of 0.55 (33). Other ergonomic interventions include improving the workplace, such as using new equipment or lifting devices, and making adjustments to the production system (31). However, interventions regarding modifications to the workplace or production system have not been sufficiently supported (31). Furthermore, an active monitoring system was necessary to evaluate the effectiveness of new musculoskeletal strategies and policies aiming at improving physical functioning and work capability (34).

On a global scale, the sex patterns of YLD were generally opposite to that of prevalence estimates. More females experienced LBP, whereas more YLD were in males. The calculation of YLD took both prevalence estimates and severity of disability into account (1). Males were more likely to undertake physically demanding work, such as construction and manufacturing, which increased the risk of work-related injuries and more severe disability. Moreover, due to differences in disease perceptions and health attitudes, compared to females, males tended to delay the utilization of routine screening and prevention and might neglect the disease until it worsened (6). This was also supported by previous findings that ≤28% of prevalent cases of LBP were in severe and most severe conditions, but contributed to 77% of LBP-associated morbidity (29, 35). Therefore, it could be expected that the majority of prevalent cases of LBP suffered from mild or moderate severity. As such, more prevalent cases were diagnosed but fewer YLD were found in females. For this reason, more health resources were utilized, and higher healthcare costs were paid by females as well. Although more severe illness might require more systematic treatments that led to higher medical costs, male workers with more severe disability generally requested a longer absence from work than female peers (6). Therefore, overall larger productivity losses and total costs were found among males.

Although >70% of the economic losses were found in high-income countries, the economic losses in low-income and middle-income countries were also a particular concern. A prior study estimated that LBP caused 59 million days of absence from work, contributing to US$1.8 billion in productivity losses between 2012 and 2016 in Brazil (6). Additionally, developing countries usually have limited financial resources to address the disability burden. However, requests for unnecessary medical examination and healthcare that contributed to long-lasting LBP-associated morbidity might co-exist with the improvement of economic conditions (34).

One suggestion for reducing the economic impact of LBP worldwide is to adopt evidence-based paradigms for healthcare practices and avoid overmedication, which often wastes scarce health resources and causes harm to patients (6). Another consideration is to seek alternative beneficial approaches and decrease reliance on medical services. Strengthening redirected disease management and problem-solving skills has been proven to be cost-effective in patients with LBP (36). This process improves patients’ coping abilities and reduces their need for medical visits by redefining LBP as a limitation in daily life instead of as a disease seeking a cure (36). Further, health policies commonly give priority to non-communicable diseases, such as cancer, cardiovascular disease, and diabetes, other than musculoskeletal disorders (37). Incorporating the prevention, treatment, and promotion of musculoskeletal health into the current local, national, and regional health policy reform will benefit the achievement of sustainable development goals (37).

### Limitations

This study had several limitations. First, for those countries without primary data, the GBD project provided modeled estimates relying on out-of-sample predictive validity (1, 8, 23, 38). For example, prevalence estimates of LBP were calculated for 204 countries and territories based on data inputs from 102 countries and territories (1). However, only slight changes in the accuracy of estimates were observed between the data samples randomly culled to 10%, 5%, 2.5%, and 1% of the original data points (1). Despite this, it is preferable to obtain primary data from each country and analyze the data with a standardized methodology. However, this is challenging, particularly in low-income countries, where representative data are sparse and data restriction regulations exist (23). Second, the labor force participation rate in each age group was missing in 84 countries and territories, which were replaced by the values from their neighboring countries after comparing countries’ income levels, distance, and aggregate labor force participation rates in people aged ≥15 years. Although the estimated results might not be consistent with reality, this was what we could do to minimize deviation. Third, our estimates were based on several assumptions. For example, we extrapolated healthcare costs per case in the US to other countries and territories with a spending ratio by assuming that variations in cross-nation disease-specific costs per case were driven completely by variations in the overall healthcare spending per head. Such extrapolation might introduce uncertainty to our results.

Additionally, LBP also had an intangible health impact, such as psychological distress, and social maladjustment (15). However, it was challenging to measure psychosocial impairment in monetary terms due to unavailable tools. Fifth, we did not include other LBP-associated costs, such as transportation costs for medication, payments for complementary and alternative therapies, and other informal professional assistance not recorded in the medical billing systems (29). For this reason, the economic impact might be underestimated in this study. Sixth, patients from different countries with various cultural backgrounds and living experiences may differ in the perception and severity of health condition, which can contribute to variations in the reporting of the disease burden across countries. However, such bias cannot be completely avoided, because variation in pain perception exists even among individuals in the same country or territory. To ensure data quality, the GBD team has made substantial efforts to data adjustments, such as corrections for bias in studies that located LBP in broad anatomical regions, and conducting adjustments with the meta-regression–Bayesian, regularized, trimmed network crosswalk adjustment approach (1). Lastly, our study is an ecological one, which may be subject to the ecological fallacy. The PAF represents the association between OEF and LBP at the population level. Therefore, conclusions based on the aggregate estimates in this study may not be inferred to individuals, who may not have the same exposure to OEF as that in the entire population.

### Concluding remarks

Globally, OEF-attributable LBP placed a considerable burden on health and economic systems in 2019. The disease and economic impact presented great variations across regions and nations. Generally, high-income countries were paying a higher price through excess healthcare costs and productivity losses, whereas middle-income countries suffered from more morbidity. Exercising with education, developing an active monitoring system, encouraging evidence-based medical practices, seeking alternative cost-effective solutions, and prioritizing musculoskeletal health in health policy reform are crucial for managing LBP worldwide. This study conducts the first investigation to examine the contribution of OEF that can provide substantial insight into softening the impact of LBP nationally, regionally, and globally.

## Supplementary material

Supplementary material

## Data Availability

The institutional review board of the University of Hong Kong/Hospital Authority Hong Kong West Cluster (UW22-131) approved this study and waived the requirement for informed consent because this study used deidentified data. All data used in this work are in public and open access repositories. Information was provided in the reference list. The authors declare no competing interests.

## References

[1] GBD 2019 Diseases and Injuries Collaborators. Global burden of 369 diseases and injuries in 204 countries and territories, 1990-2019: a systematic analysis for the Global Burden of Disease Study 2019. Lancet 2020 Oct;396(10258):1204–22. 10.1016/S0140-6736(20)30925-9 33069326 PMC7567026

[2] Chen S, Chen M, Wu X, Lin S, Tao C, Cao H et al. Global, regional and national burden of low back pain 1990-2019: A systematic analysis of the Global Burden of Disease study 2019. J Orthop Translat 2021 Sep;32:49–58. 10.1016/j.jot.2021.07.005 34934626 PMC8639804

[3] Chen N, Fong DY, Wong JY. Secular trends in musculoskeletal rehabilitation needs in 191 countries and territories from 1990 to 2019. JAMA Netw Open 2022 Jan;5(1):e2144198. 10.1001/jamanetworkopen.2021.44198 35044468 PMC8771302

[4] Dieleman JL, Cao J, Chapin A, Chen C, Li Z, Liu A et al. US health care spending by payer and health condition, 1996-2016. JAMA 2020 Mar;323(9):863–84. 10.1001/jama.2020.0734 32125402 PMC7054840

[5] Allen D, Hines EW, Pazdernik V, Konecny LT, Breitenbach E. Four-year review of presenteeism data among employees of a large United States health care system: a retrospective prevalence study. Hum Resour Health 2018 Nov;16(1):59. 10.1186/s12960-018-0321-9 30413168 PMC6234777

[6] Carregaro RL, Tottoli CR, Rodrigues DD, Bosmans JE, da Silva EN, van Tulder M. Low back pain should be considered a health and research priority in Brazil: lost productivity and healthcare costs between 2012 to 2016. PLoS One 2020 Apr;15(4):e0230902. 10.1371/journal.pone.0230902 32236113 PMC7112211

[7] Hulshof CT, Pega F, Neupane S, Colosio C, Daams JG, Kc P et al. The effect of occupational exposure to ergonomic risk factors on osteoarthritis of hip or knee and selected other musculoskeletal diseases: A systematic review and meta-analysis from the WHO/ILO Joint Estimates of the Work-related Burden of Disease and Injury. Environ Int 2021 May;150:106349. 10.1016/j.envint.2020.106349 33546919

[8] GBD 2019 Risk Factors Collaborators. Global burden of 87 risk factors in 204 countries and territories, 1990-2019: a systematic analysis for the Global Burden of Disease Study 2019. Lancet 2020 Oct;396(10258):1223–49. 10.1016/S0140-6736(20)30752-2 33069327 PMC7566194

[9] Institute for Health Metrics and Evaluation. Occupational ergonomic factors — level 3 risk. Available from: https://www.healthdata.org/results/gbd_summaries/2019/occupational-ergonomic-factors-level-3-risk

[10] Dieleman JL, Baral R, Birger M, Bui AL, Bulchis A, Chapin A et al. US spending on personal health care and public health, 1996–2013. JAMA 2016 Dec;316(24):2627–46. 10.1001/jama.2016.16885 28027366 PMC5551483

[11] Alonso-García M, Sarría-Santamera A. The economic and social burden of low back pain in Spain: a national assessment of the economic and social impact of low back pain in Spain. Spine 2020 Aug;45(16):E1026–32. 10.1097/BRS.0000000000003476 32706566

[12] Lee YR, Cho B, Jo MW, Ock M, Lee D, Lee D et al. Measuring the economic burden of disease and injury in Korea, 2015. J Korean Med Sci 2019 Mar;34 Suppl 1:e80. 10.3346/jkms.2019.34.e80 30923489 PMC6434156

[13] Olafsson G, Jonsson E, Fritzell P, Hägg O, Borgström F. Cost of low back pain: results from a national register study in Sweden. Eur Spine J 2018 Nov;27(11):2875–81. 10.1007/s00586-018-5742-6 30155730

[14] Ding D, Lawson KD, Kolbe-Alexander TL, Finkelstein EA, Katzmarzyk PT, van Mechelen W et al.; Lancet Physical Activity Series 2 Executive Committee. The economic burden of physical inactivity: a global analysis of major non-communicable diseases. Lancet 2016 Sep;388(10051):1311–24. 10.1016/S0140-6736(16)30383-X 27475266

[15] Ekman M, Johnell O, Lidgren L. The economic cost of low back pain in Sweden in 2001. Acta Orthop 2005 Apr;76(2):275–84. 10.1080/00016470510030698 16097556

[16] Institute for Health Metrics and Evaluation. GBD results tool. Available from: https://ghdx.healthdata.org/gbd-results-tool

[17] International Labour Office Department of Statistics. Statistics on labour income and inequality. Available from: https://ilostat.ilo.org/topics/labour-income/

[18] International Labour Office Department of Statistics. Employment-to-population ratio by sex and age (%) annual. Available from: https://www.ilo.org/shinyapps/bulkexplorer2/?lang=en&segment=indicator&id=EMP_DWAP_SEX_AGE_RT_A

[19] The World Bank Group. Data bank world development indicators. Available from: https://databank.worldbank.org/source/world-development-indicators

[20] International Labour Office Department of Statistics. Labour force by sex and age (thousands)-annual. Available from: https://www.ilo.org/shinyapps/bulkexplorer41/?lang=en&segment=indicator&id=EAP_TEAP_SEX_AGE_NB_A

[21] World Health Organization (WHO). WHO global health expenditure database. Available from: https://apps.who.int/nha/database/Select/Indicators/en

[22] Driscoll T, Jacklyn G, Orchard J, Passmore E, Vos T, Freedman G et al. The global burden of occupationally related low back pain: estimates from the Global Burden of Disease 2010 study. Ann Rheum Dis 2014 Jun;73(6):975–81. 10.1136/annrheumdis-2013-204631 24665117

[23] GBD 2021 Low Back Pain Collaborators. Global, regional, and national burden of low back pain, 1990-2020, its attributable risk factors, and projections to 2050: a systematic analysis of the Global Burden of Disease Study 2021. Lancet Rheumatol 2023 May;5(6):e316–29. 10.1016/S2665-9913(23)00098-X 37273833 PMC10234592

[24] Chen N, Fong DY, Wong JY. Health and economic outcomes associated with musculoskeletal disorders attributable to high body mass index in 192 countries and territories in 2019. JAMA Netw Open 2023 Jan;6(1):e2250674. 10.1001/jamanetworkopen.2022.50674 36662529 PMC9860530

[25] Global Burden of Disease 2020 Health Financing Collaborator Network. Tracking development assistance for health and for COVID-19: a review of development assistance, government, out-of-pocket, and other private spending on health for 204 countries and territories, 1990-2050. Lancet 2021 Oct;398(10308):1317–43. 10.1016/S0140-6736(21)01258-7 34562388 PMC8457757

[26] Kanal D, Kornegay JT. Accounting for household production in the national accounts: an update, 1965–2017. Available from: https://apps.bea.gov/scb/2019/06-june/0619-household-production.htm

[27] Fisher S, Bellinger DC, Cropper ML, Kumar P, Binagwaho A, Koudenoukpo JB et al. Air pollution and development in Africa: impacts on health, the economy, and human capital. Lancet Planet Health 2021 Oct;5(10):e681–8. 10.1016/S2542-5196(21)00201-1 34627472

[28] India State-Level Disease Burden Initiative Air Pollution Collaborators. Health and economic impact of air pollution in the states of India: the Global Burden of Disease Study 2019. Lancet Planet Health 2021 Jan;5(1):e25–38. 10.1016/S2542-5196(20)30298-9 33357500 PMC7805008

[29] Hartvigsen J, Hancock MJ, Kongsted A, Louw Q, Ferreira ML, Genevay S et al.; Lancet Low Back Pain Series Working Group. What low back pain is and why we need to pay attention. Lancet 2018 Jun;391(10137):2356–67. 10.1016/S0140-6736(18)30480-X 29573870

[30] Chen N, Fong DY, Wong JY. Trends in musculoskeletal rehabilitation needs in China from 1990 to 2030: a Bayesian age-period-cohort modeling study. Front Public Health 2022 Jun;10:869239. 10.3389/fpubh.2022.869239 35784203 PMC9240767

[31] Driessen MT, Proper KI, van Tulder MW, Anema JR, Bongers PM, van der Beek AJ. The effectiveness of physical and organisational ergonomic interventions on low back pain and neck pain: a systematic review. Occup Environ Med 2010 Apr;67(4):277–85. 10.1136/oem.2009.047548 20360197

[32] Asada F, Nomura T, Takano K, Kubota M, Iwasaki M, Oka T et al. Effect of quick simple exercise on non-specific low back pain in Japanese workers: a randomized controlled trial. Environ Health Prev Med 2023;28:36. 10.1265/ehpm.22-00203 37316255 PMC10287985

[33] Steffens D, Maher CG, Pereira LS, Stevens ML, Oliveira VC, Chapple M et al. Prevention of low back pain: a systematic review and meta-analysis. JAMA Intern Med 2016 Feb;176(2):199–208. 10.1001/jamainternmed.2015.7431 26752509

[34] Buchbinder R, van Tulder M, Öberg B, Costa LM, Woolf A, Schoene M et al.; Lancet Low Back Pain Series Working Group. Low back pain: a call for action. Lancet 2018 Jun;391(10137):2384–8. 10.1016/S0140-6736(18)30488-4 29573871

[35] Murray CJ, Barber RM, Foreman KJ, Abbasoglu Ozgoren A, Abd-Allah F, Abera SF et al.; GBD 2013 DALYs and HALE Collaborators. Global, regional, and national disability-adjusted life years (DALYs) for 306 diseases and injuries and healthy life expectancy (HALE) for 188 countries, 1990-2013: quantifying the epidemiological transition. Lancet 2015 Nov;386(10009):2145–91. 10.1016/S0140-6736(15)61340-X 26321261 PMC4673910

[36] Linton SJ, Boersma K, Traczyk M, Shaw W, Nicholas M. Early workplace communication and problem solving to prevent back disability: results of a randomized controlled trial among high-risk workers and their supervisors. J Occup Rehabil 2016 Jun;26(2):150–9. 10.1007/s10926-015-9596-z 26202039 PMC4854941

[37] Briggs AM, Woolf AD, Dreinhöfer K, Homb N, Hoy DG, Kopansky-Giles D et al. Reducing the global burden of musculoskeletal conditions. Bull World Health Organ 2018 May;96(5):366–8. 10.2471/BLT.17.204891 29875522 PMC5985424

[38] Maher C, Ferreira G. Time to reconsider what Global Burden of Disease studies really tell us about low back pain. Ann Rheum Dis 2022 Mar;81(3):306–8. 10.1136/annrheumdis-2021-221173 34583922 PMC8862017

