# Supplementary material for: The global health and economic impact of low-back pain attributable to occupational ergonomic factors in the working-age population by age, sex, geography in 2019
Source: Scand J Work Environ Health. 2023 Sep 29;49(7):487–96. doi: 10.5271/sjweh.4116 (PMC10838400; doi:10.5271/sjweh.4116)
Supplement: Supplementary material [file SJWEH-49-487-S001.pdf]

The global health and economic impact of low back pain attributable to occupational ergonomic factors in the working-age population by age, sex, geography in 2019[1]

by Ningjing Chen, PhD, Daniel Yee Tak Fong, PhD[2] Janet Yuen Ha Wong, PhD

1. Supplementary material
2. Correspondence to: Daniel Yee Tak Fong, PhD, School of Nursing, Li Ka Shing Faculty of Medicine, The University of Hong Kong, Hong Kong, China. [E-mail: dytfong@hku.hk]

This appendix presents methodological details and additional results for "The global health and economic impact of low back pain attributable to occupational ergonomic factors in the working-age population by age, sex, geography in 2019"

The methodological appendix has been adapted from Chen et al (1).

Table of Content

Appendix Item
Methodological Appendix
Supplementary Table S1. Sources or calculation methods of the labor force
Supplementary Table S2. Locations with missing values, substitutes, and income levels
Supplementary Table S3. Unreliable employment rates replaced in females aged 15-19 years in five countries and territories
Supplementary Table S4. A summary of variables included in this study
Supplementary Table S5. Relative risks for low back pain by age and occupation group
Supplementary Figure S1. Study selection
Supplementary Table S6. Study ranking
Supplementary Table S7. Health care costs per case of low back pain in the US
Supplementary Figure S2. Comparisons of health care costs per case (US$)
Supplementary Table S8. Disease burden of low back pain attributable to occupational ergonomic factors by location in 2019 (amount, uncertainty interval)
Supplementary Table S9. Health care costs of low back pain attributable to occupational ergonomic factors by location in 2019
Supplementary Table S10. Health care costs, productivity losses, and total costs of low back pain attributable to occupational ergonomic factors by location in 2019
Supplementary Table S11. Health care costs of low back pain attributable to occupational ergonomic factors borne by sector and location in 2019
References

Methodological Appendix

Additional information on data preparation

In 2019, the expenditure shares of health care costs paid by three sectors were not available in some countries and territories. Therefore, the most updated values of this estimate in these countries and territories were used, i.e., Albania (2018), Yemen (2015), Syria (2012), and Libya (2011). We also extracted gross domestic product (GDP) data in 2019 for Syria, Albania, and Libya from the World Bank (2), as they were not available in the World Health Organization data repository (3). Besides, we obtained Yemen's GDP in 2018 from the World Bank (2), since its GDP in 2019 was not estimated.

In addition, the total labor force in the Marshall Islands, San Marino, Kiribati, Cook Islands, and Seychelles was obtained from International Labour Organization (ILO) dataset (4), as this estimate was not provided in the World Bank data portal (2). Moreover, neither the World Bank nor the ILO dataset provided the total labor force in Grenada, Antigua and Barbuda, and Micronesia (the Federated States of). For this reason, we used the number of workers aged ≥15 to replace the total labor force in these countries and territories, which was calculated as the number of people aged ≥15 (5) multiplied by the employment rate in people aged ≥15 (6). However, the number of people aged ≥15 was not available in Dominica, Saint Kitts and Nevis, Andorra, Monaco, Palau, Nauru, and Tuvalu, where the national population size in the World Bank database (2) was used. We also extracted the national population size in Niue from its official website (7), where the latest estimate was provided in 2017 (supplementary table S1).

Additionally, in the ILO database (8), the labor income share in GDP in country i (α_i) was only available between 2004 and 2017. Since α_i was constant over this period, the mean α_i was used as the α_i for the year 2019 in country i.

Furthermore, in the ILO data repository (9), 84 countries and territories did not report the L_{ij}/N_{ij} in each age group, but only provided an aggregate L_{ij}/N_{ij} in people aged ≥15, between 15 and 24, ≥25, or ≥65. We used the L_{ij}/N_{ij} from their neighboring countries and territories to replace these missing data based on the comparisons of income levels, distance, and aggregate L_{ij}/N_{ij} in workers aged ≥15, and ≥25 (supplementary table S2). In people aged ≥85, productivity losses were assumed to be 0. Therefore, the productivity losses due to morbidity were calculated in people aged between 15 and 84 only. In people aged ≥65, only aggregate L_{ij}/N_{ij} was available. For this reason, we assumed that there was a linear decreasing trend in L_{ij}/N_{ij} from age 65 to 85, which turned to 0 at age ≥85.

Notably, five countries and territories reported unreliable employment rates in females aged 15-19 (supplementary table S3), which were replaced by the corresponding values

in neighboring countries and territories after taking into account aggregate employment rates in females aged ≥15. A summary of variables included in this study is presented in supplementary table S4.

Population attributable fraction

The population attributable fraction (PAF) describes the proportion of risk that would be reduced if the exposure to a risk factor were limited to the theoretical minimum-risk level (10). The PAF was estimated based on the comparisons of exposure categories with the reference category in each age, sex, year, and cause (10).

Specifically, in the GBD 2019 study (10), the PAF was calculated using the following formula:

$$PAF_{joasgt} = \frac{\sum_{x=1}^{u} RR_{joast}(x)P_{jasgt}(x) - RR_{joasg}(TMRE_{jas})}{\sum_{x=1}^{u} RR_{joas}(x)P_{jasgt}(x)}$$

where PAF_{joasgt} is the PAF for cause o due to risk factor j for age group a, sex s, location g, and year t. $RR_{joasg}(x)$ is the RR as a function of exposure level x for risk factor j for cause o, age group a, sex s, and location g on a plausible range of exposure levels from l to u; $P_{jasgt}(x)$ is the proportion of the population in risk group (prevalence) for age group a, sex s, location g, and year t; and $TMREL_{jas}$ is the theoretical minimum-risk exposure level (TMREL) for risk factor j, age group a, and sex s. The relative risks for LBP by age and occupation group in the GBD 2019 study (10) are shown in supplementary table S5.

Systematic review

To verify the methodological validity, due to the limited number of current national studies reporting health care costs of LBP, we expanded our search to include studies focusing on estimating health care costs of all categories of musculoskeletal disorders. We comprehensively searched the existing English-language studies in recent ten years, i.e., from January 1, 2012 to February 10, 2022. The search terms included "((cost [Title] OR spending [Title] OR expenditure [Title] OR econom*[Title]) AND (musculoskeletal [Title/Abstract] OR back pain [Title/Abstract] OR gout [Title/Abstract] OR osteoarthritis [Title/Abstract] OR neck pain [Title/Abstract] OR rheumatoid arthritis [Title/Abstract]))". The study screening process is in supplementary figure S1, as recommended by the PRISMA reporting guidelines (11).

In total, there were 1332 records. Those studies focusing on health care costs of one or more categories of musculoskeletal disorders, and comparing the health care costs between cases and controls were included. We also included those studies performing

cost-effectiveness analysis, which might also contain the original health care costs. Additional 12 records were selected from reviews. Thus, there were 1344 records, and 1338 records entered the screening process after the removal of 6 duplicates. We first screened titles and abstracts. We then screened full texts in case we were unsure about the relevance of the studies. Because studies focusing on national economic estimates were scarce, we used a broad definition for "national studies". Several estimates based on a large-scale representative sample (sample size >2000) selected from a national dataset were also included. However, we excluded those pure reviews without providing specific health care costs, studies using a sample that was not selected from a national dataset or large enough, and studies focusing on the cost-effectiveness comparisons among several strategies without estimating specific health care costs. Moreover, those studies only focusing on complication costs were also excluded, because we did not estimate the costs of complications in this study. In addition, we excluded those studies that only estimated one component of health care costs or used a sample from a subgroup (i.e., females or severe cases). We also excluded two studies that performed projection analyses or provided simulation estimates only. Therefore, 14 studies (supplementary table S6) were included and ranked based on the following criteria.

Selection criteria in descending order of importance

1. Statistical methods: Most studies performed descriptive analysis. As a patient might have multiple diseases, studies with results adjusted for comorbidities and addressed data nonrepresentativeness were given priority.
2. Precision of study results: We gave priority to those studies using observed data rather than self-reported data, which were more likely to be affected by recall, response, and social desirability bias. Additionally, misclassification of diseases might appear in self-reported data.
3. Sample size: Research findings based on a larger sample would help reduce bias. Those studies using a national sample were given priority, although the sample size was not reported.
4. Data source: The study sample should be selected from a national database. Those studies using a national sample were given priority.
5. Inclusiveness of diseases: In this study, we aimed to obtain health care costs per case for LBP. Thus, those studies that provided such estimates were given priority.
6. Year of publication: Old data might provide outdated information. Thus, those studies using the latest data were given priority. However, the bias from statistical flaws might be larger than old estimates. Therefore, we did not prioritize this benchmark.
7. Study duration: Compared to those studies using data covering one year or less, those studies using data covering longer periods would present an average level of results. However, we only considered this benchmark in case we could not draw a conclusion based on other benchmarks listed above.

Taken together, the US study was ranked first in terms of its representativeness.

Details on estimation of health care costs per case in the US

In the US study, health care costs were estimated between 1996 and 2016 only (12). We estimated health care costs in the US in 2019 by applying the annual increasing rate during this period (12). In the US study (12), health care costs of LBP and neck pain were not provided separately, and only aggregate health care spending on both LBP and neck pain was available. Therefore, we assumed that health care spending on LBP and neck pain was equal. Subsequently, we obtained the number of prevalent cases of LBP and neck pain from the GBD result tool (13). Finally, we calculated health care costs per case of LBP in the US (supplementary table S7).

Methodological validity

In our study, health care costs on LBP were estimated in each county with the extrapolation method, which assumed that variations in cross-nation disease-specific costs per case were driven completely by the variations in overall health care spending per head. Although the extrapolation approach might reduce generalizability, it has been well applied in previous economic estimations of non-communicable diseases (14-16).

So far, there have been no datasets reporting disease-specific health care costs per case. Therefore, to confirm the validity of the extrapolation method, comparisons were conducted between our estimates and research findings in previous studies (17-22). Because the prevalence estimates and per case costs were not available in two studies (17, 19), we estimated health care costs per case in these two studies by using the health care costs estimated in these two studies divided by the number of prevalent cases derived from the GBD 2019 database (13). Currency exchange rates are US$0.9879 per €1 and US$0.0068 per JPY1, as of November 5, 2022. Overall, health care costs per case estimated in our study were comparable to those in previous studies (17-22), as the points in supplementary figure S2 were near the line y = x, except for an outlier. In 2013, per case cost of LBP was US$1424.4 in the US, lower than US$2222.4 estimated in our study in 2019. The difference might be explained by the increase in health care costs per case during 2013-2016 (12), which was not considered when comparing.

Estimation of productivity losses due to morbidity

We quantified productivity losses on the basis of income per worker. To examine sex and age patterns, productivity losses were stratified by sex and age. In each sex and age group, GDP per worker was equal to the labor income share in GDP in country i (α_i) multiplied by GDP (Y_i), divided by the labor force size (L_i). Consistent with other research (23), each worker was assumed to have the same productivity in country i. Given the fact that some people at age j are unemployed, we applied a fraction of employment to the population (L_{ij}/N_{ij}) to adjust the GDP per worker at age j in country

i in 2019 (W_{ij2019}), i.e.,
$W_{ij2019} = (\alpha_i Y_i/L_i)*(L_{ij}/N_{ij})$ (1).

Subsequently, equation (1) was changed into equation (2) to include non-market production. Particularly, the contribution of non-market production (λ) was assumed to be 23% of GDP in upper-middle-income and high-income countries, and 35% of GDP in low-income and lower-middle-income countries. Therefore, the W'_{ij2019} was calculated as:
$W'_{ij2019} = (\alpha_i Y_i/L_i)*(L_{ij}/N_{ij}) + \lambda_i(\alpha_i Y_i/L_i)*[1-(L_{ij}/N_{ij})]$ (2).

In equation (2), λ_i refers to the fraction that non-market production contributes equally to GDP in country i. We used M_{ij} to present productivity losses due to morbidity, which were calculated as:

$M_{ij} = W'_{ij2019}*YLD_{ij}$ (3).

Finally, we summed M_{ij} across ages and sexes to generate total productivity losses. This method to estimate productivity losses had been used in previous studies (23, 24). We also showed the estimation process in supplementary table S4.

An example of estimation in this study

To strengthen the understanding of our estimation, we took the calculation of disease burden, health care costs, and lost output due to morbidity of LBP in the UK for example. The cited references indicate where data were derived.

1. Attributable cases and YLDs
1) Attributable cases = Cases of LBP *PAF (13)
2) Attributable YLDs: extracted from the GBD result tool (13)

2. Health care costs
1) Cost per case in the US = US spending on LBP (12)/Cases of LBP in the US (13)
The spending ratio = Overall health spending per head in the UK (25)/ Overall health spending per head in the US (25)
2) Cost per case in the UK= Cost per case in the US* the spending ratio
3) Attributable health care costs= Attributable cases* cost per case in the UK

3. Lost output due to morbidity by sex
1) The expected market production per worker of age j, i.e., $W_{j2019} = (\alpha Y/L) * (L_j/N_j)$,
α: the labor income share in GDP (8)
Y: GDP (3)
L: the number of laborers (2)
L_j/N_j: the labor force participation rate of age j, i.e., the ratio of the number of laborers (L_j) to the population size of age j (N_j) (6)

2) The market and non-market production per person, i.e.,

$W'_{ij2019} = (\alpha Y/L) * (L_j/N_j) + \lambda(\alpha Y/L) * [1-(L_j/N_j)]$

λ: The ratio of non-market production equally accounting for the UK's GDP

As the UK is a high-income country, we assumed λ to be 23%.

3) Productivity losses due to morbidity = W'_{ij2019} * Attributable YLDs

Productivity losses by sex and age were summed together to arrive at total productivity losses. The method to estimate the lost output due to morbidity has been applied in previous studies (23, 24).

Supplementary Table S1. Sources or calculation methods of the labor force

Countries and territories	Sources or calculation methods of the labor force
Marshall Islands, San Marino, Kiribati, Cook Islands, and Seychelles	The International Labour Organization database (4)
Grenada, Antigua and Barbuda, and Micronesia (the Federated States of)	The number of workers aged ≥15 was calculated as the number of people aged ≥ 15 (5) multiplied by the labor force participation rate in people aged ≥15 (6).
Dominica, Saint Kitts and Nevis, Andorra, Monaco, Palau, Nauru, and Tuvalu	The national population size extracted from the World Bank database (2) was used to multiply by the labor force participation rate in people aged ≥15 (6).
Niue	The national population size extracted from the official statistics (7) was used to multiply by the labor force participation rate in people aged ≥15 (6).
Others	The World Bank data repository (2)

Supplementary Table S2. Locations with missing values, substitutes, and income levels

Locations with missing values	Income levels of locations with missing values	Location substitutes	Income levels of location substitutes
Afghanistan	Low	Guinea	Low
Algeria	Lower-middle	Guinea	Low
Andorra	High	Spain	High
Angola	Lower-middle	Kenya	Lower-middle
Antigua and Barbuda	High	Mexico	Upper-middle
Bahamas	High	Dominican Republic	Upper-middle
Bahrain	High	Qatar	High
Bangladesh	Lower-middle	Myanmar	Lower-middle
Benin	Lower-middle	Kenya	Lower-middle
Burkina Faso	Low	Guinea	Low
Burundi	Low	Rwanda	Low
Cameroon	Lower-middle	Kenya	Lower-middle
Cape Verde	Lower-middle	Senegal	Lower-middle
Central African Republic	Low	Kenya	Lower-middle
Chad	Low	Guinea	Low
China	Upper-middle	Indonesia	Upper-middle
Comoros	Lower-middle	Rwanda	Low
Congo	Lower-middle	Rwanda	Low
Congo, Democratic Republic of the	Low	Rwanda	Low
Cuba	Upper-middle	Dominican Republic	Upper-middle
Djibouti	Lower-middle	Somalia	Low
Dominica	Upper-middle	Saint Lucia	Upper-middle
Equatorial Guinea	Upper-middle	South Africa	Upper-middle
Eritrea	Low	Somalia	Low
Eswatini	Lower-middle	South Africa	Upper-middle
Ethiopia	Low	Somalia	Low
Fiji	Upper-middle	Cook Islands	High
Gabon	Upper-middle	South Africa	Upper-middle
Gambia	Low	Guinea	Low
Ghana	Lower-middle	Guinea	Low
Grenada	Upper-middle	Saint Lucia	Upper-middle
Guinea-Bissau	Low	Guinea	Low
Haiti	Low	Guinea	Low
Iraq	Upper-middle	Jordan	Upper-middle
Kazakhstan	Upper-middle	Kyrgyzstan	Lower-middle
Kiribati	Lower-middle	Indonesia	Upper-middle
Kuwait	High	Qatar	High

Lao People's Democratic Republic	Lower-middle	Thailand	Upper-middle
Liberia	Low	Guinea	Low
Libya	Upper-middle	Guinea	Low
Madagascar	Low	Zimbabwe	Lower-middle
Malawi	Low	Zimbabwe	Lower-middle
Mali	Low	Guinea	Low
Marshall Islands	Upper-middle	Cook Islands	High
Mauritania	Lower-middle	Guinea	Low
Micronesia, the Federated States of	Lower-middle	Philippines	Lower-middle
Monaco	High	France	High
Morocco	Lower-middle	Guinea	Low
Mozambique	Low	Zimbabwe	Lower-middle
Namibia	Upper-middle	Botswana	Upper-middle
Nauru	High	Cook Islands	High
Nepal	Lower-middle	Myanmar	Lower-middle
Nicaragua	Lower-middle	Honduras	Lower-middle
Niger	Low	Guinea	Low
Niue	High	Cook Islands	High
Oman	High	Qatar	High
Palau	High	Japan	High
Papua New Guinea	Lower-middle	Indonesia	Upper-middle
Saint Kitts and Nevis	High	Mexico	Upper-middle
Saint Vincent and the Grenadines	Upper-middle	Saint Lucia	Upper-middle
Samoa	Upper-middle	Cook Islands	High
San Marino	High	Italy	High
Sao Tome and Principe	Lower-middle	South Africa	Upper-middle
Saudi Arabia	High	Qatar	High
Sierra Leone	Low	Guinea	Low
Solomon Islands	Lower-middle	Indonesia	Upper-middle
South Sudan	Low	Kenya	Lower-middle
Sudan	Low	Somalia	Low
Suriname	Upper-middle	Guyana	Upper-middle
Syrian Arab Republic	Low	Guinea	Low
Tajikistan	Low	Kyrgyzstan	Lower-middle
Tanzania, United Republic of	Lower-middle	Rwanda	Low
Timor-Leste	Lower-middle	Indonesia	Upper-middle
Togo	Low	Guinea	Low
Tonga	Upper-middle	Cook Islands	High
Trinidad and Tobago	High	Colombia	Upper-middle
Tunisia	Lower-middle	Guinea	Low
Turkmenistan	Upper-middle	Kyrgyzstan	Lower-middle

Tuvalu	Upper-middle	Cook Islands	High
Uganda	Low	Kenya	Lower-middle
Ukraine	Lower-middle	Belarus	Upper-middle
Uzbekistan	Lower-middle	Kyrgyzstan	Lower-middle
Venezuela, Bolivarian Republic of	Upper-middle	Colombia	Upper-middle
Yemen	Low	Occupied Palestinian Territory	Low

Supplementary Table S3. Unreliable employment rates replaced in females aged 15-19 years in five countries and territories

Original locations	Income levels of the original locations	Employment rates in females aged ≥15 in the original locations	Employment rates in females aged 15-19 in the original locations	Location substitutes	Income levels of location substitutes	Employment rates in females aged ≥15 in location substitutes	Employment rates in females aged 15-19 in location substitutes
Bulgaria	Upper-middle	48.34	3.32	Romania	High	44.02	5.98
Cyprus	High	52.88	5.95	Russian Federation	Upper-middle	52.91	4.25
Croatia	High	41.95	5.47	Serbia	Upper-middle	41.88	3.21
Latvia	High	52.66	6.39	Russian Federation	Upper-middle	52.91	4.25
Slovakia	High	49.18	2.62	Czechia	High	51.33	5.11

Supplementary Table S4. A summary of variables included in this study

	Generated variables	Input variables	Input variables accompanied by 95% uncertainty intervals	Input data sources	Calculation methods or extraction
Disease burden	a. Attributable cases	a1. Cases of LBP<br>a2. PAFs	a1 and a2	GBD result tool (13)	a = a1*a2
	-	b. Attributable YLDs	b	GBD result tool (13)	Extracted from the GBD result tool (13)
Health care costs	c. Costs per case in the US	c1. Costs of LBP in the US	-	The US study (12)	c = c1/c2
		c2. Cases of LBP in the US	-	GBD result tool (13)	
	d. Costs per case in each country	Overall health spending per capita in each country	-	The global health spending study (25)	The spending ratio = Overall health spending per capita in a country/Overall health spending per capita in the US<br><br>d = c*the spending ratio
	e. Attributable health care costs in each country	d and a	Listed above	Listed above	e = d*a
	f. Attributable health care costs by sector	Expenditure shares	-	The WHO Global Health Expenditure Database (3)	f = e *expenditure shares
Productivity losses		g1. The labor income share in GDP in country *i* (α_i)	-	ILO database (8)	Extracted from the ILO database (8)

	g. The lost market output per worker by sex	g2. GDP (Y_i)	-	The WHO Global Health Expenditure Database (3), and the World Bank (2)	Extracted from the WHO Global Health Expenditure Database (3), and the World Bank (2)
		g3. The number of laborers (L_i)	-	The World Bank database (2), and the ILO data repository (4)	The L_i in several countries and territories was calculated based on the labor force participation rate in people aged 15 and older (supplementary table S1) (6).
		g4. The ratio of the number of laborers to the population size of age j (L_{ij}/N_{ij})	-	ILO websites (6)	We estimated the lost output per worker of age j by sex (W_{ij2019}) as follows- $W_{ij2019} = (\alpha_i Y_i/L_i)*(L_{ij}/N_{ij})$.
	h. The lost non-market output per person by sex	The ratio of non-market production equally accounting for GDP in country i (λ_i)	-	The official website of the US government (26) and a previous study (23)	λ_i was assumed to be 23% in high-income and upper-middle-income countries, and 35% in lower-middle-income and low-income countries.<br><br>The lost market and non-market output per person by sex, i.e., $W'_{ij2019} = (\alpha_i Y_i/L_i)*(L_{ij}/N_{ij}) + \lambda_i(\alpha_i Y_i/L_i)*[1-(L_{ij}/N_{ij})]$.

Abbreviations: GBD, Global Burden of Diseases, Injuries, and Risk Factors Study; GDP, gross domestic product; ILO, International Labour Organization; LBP, low back pain; PAFs, population attributable fractions; WHO, World Health Organization; YLDs, years lived with disability

Supplementary Table S5. Relative risks for low back pain by age and occupation group

	Age (years)													
Occupation groups	15-19	20-24	25-29	30-34	35-39	40-44	45-49	50-54	55-59	60-64	65-69	70-74	75-79	80-84
Professional, technical and related workers	1.173 (1.066 to 1.282)	1.172 (1.062 to 1.283)	1.169 (1.065 to 1.283)	1.17 (1.062 to 1.284)	1.17 (1.062 to 1.285)	1.172 (1.062 to 1.283)	1.171 (1.071 to 1.27)	1.169 (1.063 to 1.288)	1.171 (1.059 to 1.281)	1.17 (1.058 to 1.286)	1.17 (1.07 to 1.279)	1.172 (1.065 to 1.287)	1.172 (1.07 to 1.283)	1.172 (1.07 to 1.283)
Administrative and managerial workers	1.211 (0.964 to 1.508)	1.21 (0.964 to 1.492)	1.209 (0.965 to 1.487)	1.209 (0.963 to 1.524)	1.207 (0.976 to 1.496)	1.207 (0.965 to 1.5)	1.205 (0.946 to 1.489)	1.205 (0.967 to 1.472)1.0	1.205 (0.961 to 1.509)	1.203 (0.948 to 1.515)	1.209 (0.976 to 1.479)	1.21 (0.964 to 1.49)	1.203 (0.961 to 1.501)	1.203 (0.961 to 1.501)
Clerical and related workers	1.0 (1.0 to 1.0)	1.0 (1.0 to 1.0)	1.0 (1.0 to 1.0)	1.0 (1.0 to 1.0)	1.0 (1.0 to 1.0)	1.0 (1.0 to 1.0)	1.0 (1.0 to 1.0)	1.0 (1.0 to 1.0)	1.0 (1.0 to 1.0)	1.0 (1.0 to 1.0)	1.0 (1.0 to 1.0)	1.0 (1.0 to 1.0)	1.0 (1.0 to 1.0)	1.0 (1.0 to 1.0)
Sales workers	1.22 (1.029 to 1.434)	1.21 (1.018 to 1.418)	1.213 (1.028 to 1.434)	1.214 (1.004 to 1.448)	1.207 (1.017 to 1.445)	1.218 (1.016 to 1.455)	1.212 (1.012 to 1.444)	1.216 (1.01 to 1.448)	1.219 (1.019 to 1.45)	1.211 (1.014 to 1.444)	1.213 (1.015 to 1.455)	1.21 (1.007 to 1.423)	1.214 (1.017 to 1.446)	1.214 (1.017 to 1.446)
Service workers	1.472 (1.385 to 1.568)	1.472 (1.383 to 1.569)	1.471 (1.372 to 1.563)	1.472 (1.382 to 1.571)	1.469 (1.378 to 1.567)	1.472 (1.377 to 1.57)	1.469 (1.375 to 1.568)	1.47 (1.378 to 1.57)	1.472 (1.379 to 1.575)	1.472 (1.381 to 1.572)	1.474 (1.386 to 1.571)	1.47 (1.377 to 1.568)	1.472 (1.38 to 1.571)	1.472 (1.38 to 1.571)
Agriculture, animal husbandry and forestry workers, fishermen and hunters	3.789 (2.58 to 5.376)	3.762 (2.621 to 5.284)	3.869 (2.642 to 5.486)	3.775 (2.569 to 5.369)	3.774 (2.606 to 5.314)	3.771 (2.532 to 5.317)	3.793 (2.632 to 5.361)	3.785 (2.556 to 5.333)	3.776 (2.645 to 5.157)	3.792 (2.536 to 5.421)	3.802 (2.684 to 5.428)	3.746 (2.609 to 5.175)	3.77 (2.635 to 5.151)	3.77 (2.635 to 5.151)
Production and related workers, transport	1.543 (1.409 to 1.679)	1.54 (1.406 to 1.676)	1.542 (1.415 to 1.677)	1.543 (1.413 to 1.695)	1.542 (1.416 to 1.685)	1.543 (1.418 to 1.685)	1.541 (1.402 to 1.684)	1.542 (1.41 to 1.684)	1.541 (1.404 to 1.683)	1.54 (1.414 to 1.677)	1.54 (1.408 to 1.683)	1.538 (1.408 to 1.673)	1.541 (1.41 to 1.677)	1.541 (1.41 to 1.677)

equipment operators and laborers														

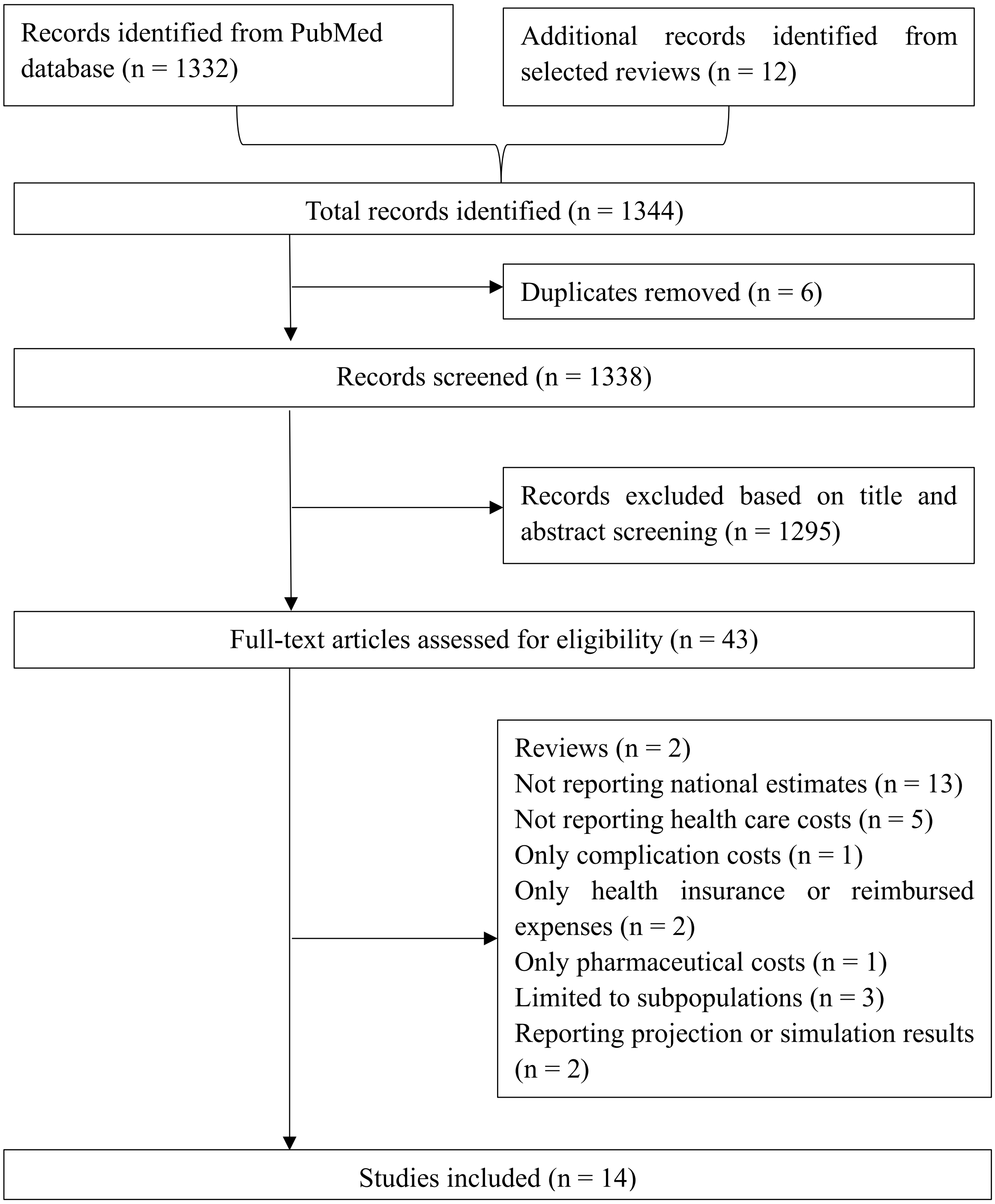

Supplementary Figure S1. Study selection

Supplementary Table S6. Study ranking

Rank	Reference	Year of publication	Country	Data sources	Sample size	Statistical methods	Precision of study results	Inclusiveness of diseases	Study duration
1	Dieleman et al (12)	2020	The US	Government budgets, insurance claims, facility records, household surveys, and official US records.	A national sample. The sample size was not provided.	Spending estimates were adjusted for comorbidities. Data nonrepresentativeness was addressed and specific adjustments were discussed.	Samples were identified based on the diagnosis code.<br><br>The degree to which the parameters were constrained was determined.<br><br>The confidence intervals were calculated.	Low back, neck pain, osteoarthritis, gout, rheumatoid arthritis, and other MDs.	1996-2016
2	Dieleman et al (17)	2016	The US	Government budgets, insurance claims, facility surveys, household surveys, and official US records.	A national sample. The sample size was not provided.	Spending was adjusted to reflect the health condition treated.	In some cases, a small number of cases were used as a basis for estimation.<br><br>The uncertainty intervals were calculated.	Low back, neck pain, osteoarthritis, gout, rheumatoid arthritis, and other MDs.	1996-2013

3	Lee et al (19)	2019	Korea	National Health Insurance claims data.	Nationally representati-ve sample. The sample size was not provided.	The economic burden was estimated with a human resources approach.	Not very precise because patients were defined based on their use of health care services, but there is a possibility that the number of patients was under- or overestimated.	LBP and osteoarthritis.	2015
4	Ahn et al (27)	2016	Korea	The sample was selected from the Korean National Health Insurance claims database.	The sample size for patients with LBP was 111544.	Descriptive analysis.	Definitions of LBP and lumbar disorders relying solely on claims of disease diagnosis have limited accuracy.	LBP, intervertebral disc disorder, and spinal stenosis.	2011
5	Olafsson et al (20)	2018	Swe-den	The sample was selected from national registers and an administrative patient database.	129,973	Using a prevalence-based bottom-up approach.	Descriptive statistics accompanied by 95% confidence interval<br><br>Limit to a region of Sweden<br><br>Several costs were not included (e.g.,	LBP	2008-2011

							out-of-pocket costs for over-the-counter drugs).		
6	Alonso-García and Sarría-Santamera (22)	2020	Spain	A nationally representative sample.	23,089	Descriptive analysis.	Not very precise because the information on LBP was self-reported.<br><br>Costs with missing values were imputed.	LBP	2017
7	Ebata-Kogure et al (18)	2020	Japan	The sample was selected from a medical claims database.	1,006,385	Descriptive analysis.	Not very precise because the costs were included regardless of whether they were incurred for hip or knee osteoarthritis or other concurrent diseases.<br><br>Uncertainty intervals were not estimated, but the interquartile ranges of health care costs were	Hip and knee osteoarthritis.	2013-2019

							estimated.		
8	Zhao et al (21)	2019	The US	Weighted nationally representative data from the 2015 Medical Expenditure Panel Survey	25,562,623	Rao-Scott chi-square tests for categorical variables and t-tests for continuous variables.	Using self-reported data	Osteoarthritis	2015
9	Flores et al (28)	2019	The US	National Health and Wellness Survey	2559	Descriptive analysis based on self-reported gout diagnosis and symptoms.	Using self-reported data	Gout	2012-2013
10	Williams et al (29)	2017	The US	The Medical Expenditures Panel Survey	35,313	Descriptive analysis.	Spending adjusted for functional limitations.<br><br>Based on self-report data.	Arthritis and joint pain	2011
11	Eriksson et al (30)	2015	Swe-den	Collected from national registries.<br><br>Costs data were derived from several sources.	49,829	Descriptive analysis.	The costs were underestimated due to the unavailable information on some cost components.<br><br>The uncertainty intervals were	Rheumatoid arthritis	2010

							calculated.		
12	Kalkan et al (31)	2014	Swe-den	Collected from national and regional registries.	Ranged from 2539 to 8058 annually.	Descriptive analysis.	Missing data were estimated based on assumptions.<br><br>Data for outpatient care and sick leave were not complete for all years in the 1990s, and average values were used, leading to possible over- or underestimation of minor importance for the total costs.	Rheumatoid arthritis	1990-2010
13	Huscher et al (32)	2015	Ger-man	Collected from the National Database of the German Collaborative Arthritis Centers.	About 3400 patients annually.	Costs were calculated using fixed prices as well as annually updated cost factors.	The authors probably underestimated 12-months related components (i.e., medication costs) due to memory bias.	Rheumatoid arthritis	2002-2011
14	Kinge et al (33)	2017	Nor-way	The Norwegian Directorate of Health.	The sample size was not provided.	Descriptive analysis.	This study did not provide the estimates we need because only an	Musculoskele-tal system	2013

							aggregate cost for the musculoskeletal system was available.		

Abbreviations: LBP, low back pain; MDs, musculoskeletal disorders.

Supplementary Table S7. Health care costs per case of low back pain in the US

Health care costs, 2016 US$ billion[a]	Annualized rate of change, 1996–2016 (%)[a]	Health care costs, 2019 US$ billion	Prevalence, 2019[b]	Health care costs per case, 2019 US$
95.6	6.6	115.8	52,105,428	2222.4

[a] Based on estimates from Dieleman et al (12)

[b] Based on estimates from the GBD 2019 Diseases and Injuries Collaborators (34)

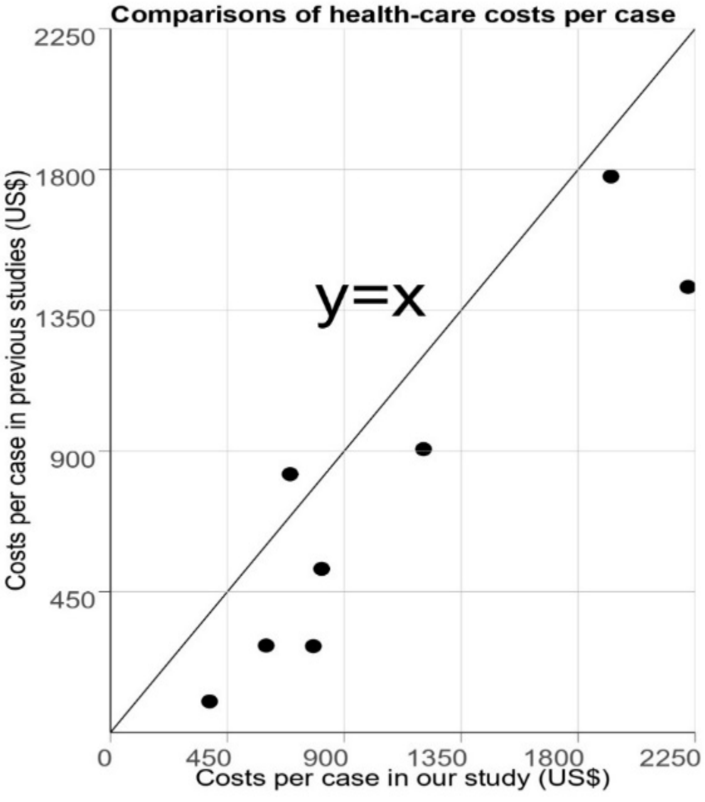

Supplementary Figure S2. Comparisons of health care costs per case (US$)

Supplementary Table S8. Disease burden of low back pain attributable to occupational ergonomic factors by location in 2019

Location	Prevalence	Years lived with disability
Global	126124974 (81613636 to 187779735)	15127747 (8710017 to 24140739)
Africa	16152306 (10327489 to 24216741)	1998745 (1129834 to 3225302)
Algeria	417422 (261882 to 643395)	54169 (30411 to 87342)
Angola	348786 (216105 to 540115)	42939 (23683 to 70549)
Benin	208554 (132544 to 317886)	25690 (14411 to 41751)
Botswana	28607 (17886 to 44113)	3633 (2032 to 5916)
Burkina Faso	362387 (229415 to 545509)	46094 (25755 to 74842)
Burundi	221216 (143224 to 328229)	27095 (15632 to 43139)
Cabo Verde	9260 (5743 to 14390)	1208 (663 to 1980)
Cameroon	592027 (383442 to 879337)	73142 (41243 to 117992)
Central African Republic	78533 (48325 to 121818)	9562 (5092 to 15957)
Chad	262221 (165044 to 392474)	32012 (17346 to 52564)
Comoros	16210 (10254 to 24543)	1994 (1106 to 3249)
Congo	53298 (33190 to 82419)	6670 (3655 to 11027)
Democratic Republic of the Congo	1253831 (779591 to 1932711)	151655 (84720 to 247868)
Equatorial Guinea	12121 (7482 to 18935)	1451 (783 to 2414)
Eritrea	73848 (46646 to 110301)	9376 (5239 to 15258)
Eswatini	7851 (4881 to 12124)	974 (552 to 1583)
Ethiopia	1168038 (744017 to 1752508)	148684 (84116 to 239943)
Gabon	14748 (9155 to 22898)	1847 (1007 to 3047)
Gambia	26816 (16618 to 41192)	3351 (1820 to 5556)
Ghana	467114 (306020 to 687844)	58612 (33660 to 93248)
Guinea	225194 (143895 to 337337)	27678 (15033 to 45634)
Guinea-Bissau	29770 (18540 to 45208)	3728 (2027 to 6159)
Ivory Coast	412497 (258935 to 631030)	52459 (29187 to 85928)
Kenya	867751 (557416 to 1296483)	107525 (61551 to 171884)
Lesotho	19868 (12536 to 30601)	2430 (1354 to 3962)
Liberia	79428 (50513 to 120470)	9912 (5593 to 15989)
Madagascar	510785 (331695 to 755479)	64397 (36964 to 102900)
Malawi	271479 (169804 to 413907)	33546 (18765 to 54525)
Mali	300211 (191456 to 453099)	37989 (21075 to 62099)
Mauritania	49561 (30474 to 76645)	6159 (3226 to 10428)
Mauritius	20319 (13145 to 30313)	2402 (1397 to 3819)
Mozambique	576786 (376050 to 848036)	69242 (39445 to 110688)
Namibia	32399 (20343 to 50060)	3951 (2211 to 6450)
Niger	367300 (236786 to 543839)	46390 (26271 to 74679)
Nigeria	3279662 (2121085 to 4855810)	400531 (228654 to 642150)

Rwanda	298869 (194163 to 441180)	36551 (20889 to 58438)
Sao Tome and Principe	1590 (978 to 2500)	203 (101 to 351)
Senegal	189639 (117480 to 290073)	23823 (12969 to 39383)
Seychelles	1900 (1218 to 2874)	244 (140 to 390)
Sierra Leone	132460 (84150 to 198453)	16108 (9063 to 26200)
South Africa	369156 (236005 to 555266)	46771 (27235 to 73951)
South Sudan	155630 (99069 to 235085)	18984 (10574 to 30788)
Togo	131036 (82900 to 201202)	16757 (9376 to 27485)
Uganda	702142 (452338 to 1039029)	86829 (48750 to 140051)
United Republic of Tanzania	885402 (565128 to 1332266)	108864 (62500 to 174540)
Zambia	313839 (202081 to 466861)	37752 (21375 to 61032)
Zimbabwe	304742 (197839 to 450894)	37361 (21185 to 60171)
The Americas	18985664 (13107452 to 26802696)	2176086 (1300371 to 3366057)
Antigua and Barbuda	1106 (704 to 1680)	136 (74 to 227)
Argentina	612845 (393821 to 918737)	71710 (41161 to 113511)
Bahamas	4127 (2639 to 6192)	516 (284 to 848)
Barbados	4282 (2744 to 6457)	487 (276 to 783)
Belize	4806 (3062 to 7209)	604 (346 to 964)
Bolivia (Plurinational State of)	222823 (140375 to 337542)	27188 (15332 to 43883)
Brazil	3926100 (2499701 to 5889276)	479369 (276339 to 765090)
Canada	663430 (430478 to 981447)	71118 (41026 to 112748)
Chile	278377 (179009 to 416043)	33053 (19103 to 52638)
Colombia	743466 (489227 to 1090746)	89658 (51756 to 143358)
Costa Rica	58621 (38012 to 88081)	7164 (4137 to 11410)
Cuba	134334 (92427 to 189978)	15079 (8601 to 23926)
Dominica	1022 (635 to 1578)	119 (63 to 200)
Dominican Republic	132979 (85596 to 198722)	16442 (9438 to 26275)
Ecuador	243103 (168939 to 343638)	29644 (17761 to 45547)
El Salvador	88082 (57070 to 130966)	10511 (6018 to 16797)
Grenada	1498 (948 to 2296)	182 (101 to 297)
Guatemala	252114 (163320 to 376445)	30946 (17695 to 49589)
Guyana	8009 (5079 to 12185)	995 (557 to 1613)
Haiti	156731 (96476 to 250200)	19186 (9827 to 32908)
Honduras	128695 (82381 to 193186)	15698 (8946 to 25251)
Jamaica	42183 (26690 to 64268)	5114 (2874 to 8264)
Mexico	1580807 (1020792 to 2344997)	191874 (111193 to 302933)
Nicaragua	98357 (63177 to 146638)	12219 (6838 to 19921)
Panama	61182 (39093 to 92604)	7393 (4209 to 11913)
Paraguay	136436 (88084 to 204165)	16874 (9739 to 26929)
Peru	506972 (329731 to 756265)	62198 (36167 to 98958)
Saint Kitts and Nevis	760 (477 to 1160)	95 (51 to 157)
Saint Lucia	2816 (1756 to 4353)	340 (188 to 553)

Saint Vincent and the Grenadines	1369 (866 to 2098)	163 (89 to 270)
Suriname	6251 (3978 to 9420)	761 (435 to 1225)
Trinidad and Tobago	17654 (11240 to 26431)	2064 (1173 to 3324)
United States of America	8468703 (6335259 to 11116087)	909325 (571583 to 1346842)
Uruguay	58053 (37174 to 86115)	6441 (3702 to 10178)
Venezuela (Bolivarian Republic of)	337568 (216491 to 505493)	41420 (23287 to 66729)
Eastern Mediterranean	8359954 (5192932 to 12933545)	1079559 (594253 to 1778334)
Afghanistan	435522 (267634 to 675393)	56699 (30687 to 94778)
Bahrain	20990 (13508 to 31386)	2954 (1703 to 4712)
Djibouti	15458 (9396 to 24187)	2002 (1033 to 3400)
Egypt	1276463 (811655 to 1941197)	162481 (90557 to 265017)
Iran (Islamic Republic of)	1214740 (787147 to 1814987)	158433 (91121 to 253203)
Iraq	304370 (194038 to 457367)	39301 (22249 to 62856)
Jordan	75682 (47287 to 116263)	10267 (5856 to 16580)
Kuwait	65572 (42015 to 98088)	9348 (5339 to 14934)
Lebanon	45737 (28982 to 69386)	5528 (3023 to 9125)
Libya	66348 (40736 to 103383)	8981 (4773 to 15047)
Morocco	702333 (437104 to 1079492)	87313 (47284 to 144556)
Oman	55510 (34670 to 85466)	8453 (4821 to 13475)
Pakistan	2310281 (1366561 to 3719840)	292881 (154592 to 497405)
Qatar	53058 (34581 to 77933)	7593 (4421 to 11934)
Saudi Arabia	489834 (313085 to 731605)	70671 (41166 to 111313)
Sudan	465491 (286952 to 730111)	56800 (30000 to 96246)
Syrian Arab Republic	145478 (89840 to 227030)	17687 (9633 to 29721)
Tunisia	147811 (91909 to 232063)	18096 (10111 to 29683)
United Arab Emirates	179520 (116031 to 269468)	27248 (15813 to 42904)
Yemen	289757 (179801 to 448900)	36823 (20068 to 61445)
Europe	19032080 (12364480 to 28231025)	2125046 (1231135 to 3365204)
Albania	106587 (68372 to 159680)	12213 (6821 to 19637)
Andorra	1669 (1052 to 2512)	199 (108 to 328)
Armenia	87325 (55310 to 131494)	10589 (5937 to 17126)
Austria	167160 (107421 to 249425)	17541 (10165 to 27759)
Azerbaijan	225593 (142796 to 342473)	30296 (17123 to 48902)
Belarus	193389 (124325 to 290585)	21633 (12239 to 34722)
Belgium	185220 (119077 to 275713)	19618 (11377 to 30952)
Bosnia and Herzegovina	67165 (42087 to 103717)	7320 (4163 to 11830)
Bulgaria	165456 (107330 to 244402)	17736 (10385 to 27734)
Croatia	104535 (66366 to 158577)	10860 (6220 to 17331)
Cyprus	26065 (17012 to 38189)	3059 (1780 to 4809)
Czechia	250137 (163968 to 369404)	27213 (16043 to 42510)

Denmark	144926 (94074 to 214473)	15278 (8816 to 24158)
Estonia	25948 (17026 to 38313)	2819 (1641 to 4441)
Finland	110513 (73031 to 161701)	11083 (6438 to 17515)
France	1244801 (808453 to 1838044)	129803 (75578 to 203987)
Georgia	87624 (54093 to 136632)	10336 (5786 to 16834)
Germany	1933070 (1296453 to 2793594)	205228 (121656 to 318860)
Greece	233909 (146529 to 360765)	24738 (13859 to 39883)
Hungary	244272 (160294 to 360771)	26010 (15293 to 40431)
Iceland	8951 (5913 to 13002)	1037 (604 to 1637)
Ireland	98665 (65156 to 143960)	11247 (6558 to 17668)
Israel	141817 (92305 to 209179)	16415 (9370 to 26107)
Italy	1101525 (716805 to 1629673)	117429 (68837 to 183305)
Kazakhstan	316555 (200424 to 480603)	40540 (23168 to 65087)
Kyrgyzstan	111394 (69885 to 169156)	14788 (8290 to 23988)
Latvia	40812 (26470 to 60395)	4300 (2513 to 6799)
Lithuania	64060 (41526 to 95813)	6862 (3980 to 10806)
Luxembourg	9693 (6258 to 14386)	1122 (649 to 1766)
Malta	9236 (6120 to 13493)	910 (530 to 1426)
Monaco	681 (437 to 1012)	67 (38 to 107)
Montenegro	12550 (8018 to 19002)	1414 (808 to 2262)
Netherlands	309941 (200687 to 454103)	32488 (18705 to 51552)
North Macedonia	36193 (23421 to 53948)	4241 (2472 to 6679)
Norway	105477 (69143 to 153727)	11703 (6787 to 18334)
Poland	1050868 (690028 to 1550442)	116034 (67821 to 181490)
Portugal	301813 (199746 to 441038)	31857 (18773 to 49937)
Republic of Moldova	73120 (46153 to 112704)	8727 (4957 to 14093)
Romania	690343 (439898 to 1043237)	76539 (43729 to 122456)
Russian Federation	2797912 (1821295 to 4128248)	313119 (184203 to 493435)
San Marino	711 (448 to 1081)	77 (45 to 122)
Serbia	261118 (165500 to 395870)	28060 (15925 to 45019)
Slovakia	113759 (73836 to 167967)	12929 (7543 to 20217)
Slovenia	47355 (30482 to 70932)	5001 (2906 to 7916)
Spain	662887 (427163 to 995242)	73942 (42974 to 117676)
Sweden	200153 (131614 to 292180)	20984 (12238 to 32851)
Switzerland	225069 (147718 to 328509)	24743 (14399 to 38798)
Tajikistan	130311 (80356 to 204516)	17316 (8962 to 29662)
Turkey	1532585 (971841 to 2322722)	188041 (106004 to 305852)
Turkmenistan	78198 (47731 to 122996)	10340 (5400 to 17496)
Ukraine	817132 (533670 to 1207187)	89630 (51884 to 141471)
United Kingdom	1420350 (947702 to 2052059)	151762 (89530 to 236683)

Uzbekistan	655482 (411664 to 1012177)	87809 (49103 to 142757)
Southeast Asia	29211205 (18763165 to 43939426)	3586462 (2062403 to 5736188)
Bangladesh	2631646 (1648882 to 4058204)	322638 (178648 to 529698)
Bhutan	20478 (13229 to 30395)	2518 (1423 to 4050)
India	14999089 (9612079 to 22605378)	1811808 (1048160 to 2889624)
Indonesia	6838649 (4476968 to 10157877)	868323 (504533 to 1376058)
Maldives	7333 (4620 to 11238)	1034 (584 to 1665)
Myanmar	1083387 (676400 to 1672536)	138605 (77436 to 225062)
Nepal	1002592 (649891 to 1472264)	121843 (69964 to 193841)
Sri Lanka	422933 (271615 to 639534)	51005 (28986 to 82351)
Thailand	2186076 (1397708 to 3262552)	266431 (151458 to 430038)
Timor-Leste	19021 (11773 to 29448)	2257 (1210 to 3801)
Western Pacific	34383765 (21858117 to 51656303)	4161850 (2392021 to 6669655)
Australia	456506 (295458 to 672270)	49672 (28636 to 78607)
Brunei Darussalam	5803 (3713 to 8657)	776 (438 to 1232)
Cambodia	506949 (331633 to 747877)	64333 (36809 to 102085)
China	23288737 (14602871 to 35311453)	2859880 (1632781 to 4616639)
Cook Islands	374 (241 to 560)	43 (25 to 69)
Fiji	9440 (6091 to 14028)	1185 (659 to 1925)
Japan	3471964 (2288493 to 5056641)	345160 (203384 to 539308)
Kiribati	1246 (769 to 1954)	163 (86 to 274)
Lao People's Democratic Republic	199586 (128025 to 296445)	25916 (14567 to 41715)
Malaysia	493414 (316476 to 742943)	62972 (36090 to 100842)
Marshall Islands	530 (330 to 819)	69 (36 to 117)
Micronesia (the Federated States of)	1410 (887 to 2148)	181 (102 to 293)
Mongolia	54201 (33794 to 83423)	7598 (4279 to 12245)
Nauru	128 (81 to 195)	17 (10 to 28)
New Zealand	99349 (63917 to 149410)	10898 (6254 to 17383)
Niue	28 (18 to 42)	3 (2 to 5)
Palau	354 (225 to 532)	45 (26 to 72)
Papua New Guinea	138751 (85971 to 216336)	18116 (9699 to 30388)
Philippines	1962867 (1301579 to 2858820)	247339 (145437 to 387646)
Republic of Korea	1234289 (795398 to 1833280)	148737 (86751 to 235627)
Samoa	3013 (1882 to 4657)	375 (201 to 635)

Singapore	72210 (45989 to 108084)	9092 (5143 to 14558)
Solomon Islands	11144 (7024 to 17178)	1422 (789 to 2314)
Tonga	1423 (888 to 2182)	175 (88 to 300)
Tuvalu	182 (114 to 275)	22 (12 to 36)
Vanuatu	8783 (5711 to 13057)	1074 (601 to 1731)
Viet Nam	2361085 (1540538 to 3513036)	306585 (179116 to 483580)

Values in parentheses represent uncertainty intervals derived from the sensitivity analyses.

Supplementary Table S9. Health care costs of low back pain attributable to occupational ergonomic factors by location in 2019

Location	Health care costs per capita, 2019, $PPP	Spending ratio	Health care costs per case, 2019, US$	Prevalence cases	Health care costs, US$
Global	3073	0.271	602	126124974 (81613636 to 187779735)	46982354312 (32326654377 to 66462804489)
Africa	318	0.028	62	16152306 (10327489 to 24216741)	581160977 (371499053 to 872957890)
Algeria	765	0.067	150	417422 (261882 to 643395)	62554415 (39245371 to 96418510)
Angola	195	0.017	38	348786 (216105 to 540115)	13323399 (8255074 to 20632038)
Benin	79	0.007	15	208554 (132544 to 317886)	3227505 (2051199 to 4919489)
Botswana	1144	0.101	224	28607 (17886 to 44113)	6410946 (4008263 to 9885909)
Burkina Faso	119	0.010	23	362387 (229415 to 545509)	8447740 (5347992 to 12716563)
Burundi	86	0.008	17	221216 (143224 to 328229)	3726806 (2412882 to 5529638)
Cabo Verde	380	0.033	74	9260 (5743 to 14390)	689329 (427511 to 1071197)
Cameroon	121	0.011	24	592027 (383442 to 879337)	14032913 (9088802 to 20843079)
Central African Republic	61	0.005	12	78533 (48325 to 121818)	938430 (577461 to 1455664)
Chad	71	0.006	14	262221 (165044 to 392474)	3647090 (2295507 to 5458716)
Comoros	187	0.016	37	16210 (10254 to 24543)	593793 (375626 to 899052)
Congo	93	0.008	18	53298 (33190 to 82419)	970996 (604669 to 1501526)

Democratic Republic of the Congo	43	0.004	8	1253831 (779591 to 1932711)	10561576 (6566836 to 16280075)
Equatorial Guinea	554	0.049	109	12121 (7482 to 18935)	1315448 (812022 to 2054888)
Eritrea	46	0.004	9	73848 (46646 to 110301)	665457 (420333 to 993937)
Eswatini	593	0.052	116	7851 (4881 to 12124)	911958 (567037 to 1408397)
Ethiopia	75	0.007	15	1168038 (744017 to 1752508)	17160865 (10931141 to 25747933)
Gabon	541	0.048	106	14748 (9155 to 22898)	1563006 (970225 to 2426746)
Gambia	118	0.010	23	26816 (16618 to 41192)	619854 (384138 to 952169)
Ghana	189	0.017	37	467114 (306020 to 687844)	17294429 (11330090 to 25466718)
Guinea	124	0.011	24	225194 (143895 to 337337)	5470158 (3495338 to 8194201)
Guinea-Bissau	178	0.016	35	29770 (18540 to 45208)	1038037 (646462 to 1576368)
Ivory Coast	170	0.015	33	412497 (258935 to 631030)	13736967 (8623053 to 21014540)
Kenya	248	0.022	49	867751 (557416 to 1296483)	42156843 (27080234 to 62985375)
Lesotho	390	0.034	76	19868 (12536 to 30601)	1517914 (957746 to 2337898)
Liberia	154	0.014	30	79428 (50513 to 120470)	2396147 (1523861 to 3634300)
Madagascar	70	0.006	14	510785 (331695 to 755479)	7004180 (4548387 to 10359565)
Malawi	107	0.009	21	271479 (169804 to 413907)	5690382 (3559210 to 8675764)
Mali	80	0.007	16	300211 (191456 to 453099)	4704766 (3000400 to 7100749)
Mauritania	210	0.019	41	49561 (30474 to 76645)	2038833 (1253620 to 3153001)
Mauritius	1553	0.137	304	20319 (13145 to 30313)	6181522 (3999015 to 9221831)

Mozambique	100	0.009	20	576786 (376050 to 848036)	11298887 (7366602 to 16612521)
Namibia	964	0.085	189	32399 (20343 to 50060)	6118347 (3841542 to 9453429)
Niger	71	0.006	14	367300 (236786 to 543839)	5108588 (3293329 to 7563972)
Nigeria	192	0.017	38	3279662 (2121085 to 4855810)	123353497 (79777497 to 182634997)
Rwanda	153	0.013	30	298869 (194163 to 441180)	8957633 (5819406 to 13222950)
Sao Tome and Principe	234	0.021	46	1590 (978 to 2500)	72881 (44814 to 114606)
Senegal	158	0.014	31	189639 (117480 to 290073)	5869557 (3636163 to 8978107)
Seychelles	1440	0.127	282	1900 (1218 to 2874)	535922 (343546 to 810643)
Sierra Leone	241	0.021	47	132460 (84150 to 198453)	6253513 (3972762 to 9369040)
South Africa	1202	0.106	235	369156 (236005 to 555266)	86923160 (55570868 to 130745398)
South Sudan	83	0.007	16	155630 (99069 to 235085)	2530424 (1610783 to 3822302)
Togo	96	0.008	19	131036 (82900 to 201202)	2464240 (1559001 to 3783762)
Uganda	131	0.012	26	702142 (452338 to 1039029)	18018445 (11607949 to 26663675)
United Republic of Tanzania	103	0.009	20	885402 (565128 to 1332266)	17864829 (11402641 to 26881231)
Zambia	205	0.018	40	313839 (202081 to 466861)	12603245 (8115227 to 18748321)
Zimbabwe	211	0.019	41	304742 (197839 to 450894)	12596103 (8177413 to 18637097)
The Americas	7659	0.675	1500	18985664 (13107452 to 26802696)	22081723424 (16183093500 to 29556474057)
Antigua and Barbuda	1281	0.113	251	1106 (704 to 1680)	277483 (176783 to 421464)

Argentina	2285	0.201	448	612845 (393821 to 918737)	274320205 (176281161 to 411243193)
Bahamas	2468	0.218	483	4127 (2639 to 6192)	1995173 (1276008 to 2993790)
Barbados	1020	0.090	200	4282 (2744 to 6457)	855643 (548360 to 1290170)
Belize	431	0.038	84	4806 (3062 to 7209)	405814 (258545 to 608616)
Bolivia (Plurinational State of)	585	0.052	115	222823 (140375 to 337542)	25535039 (16086690 to 38681597)
Brazil	1443	0.127	283	3926100 (2499701 to 5889276)	1109810364 (706603046 to 1664751259)
Canada	5837	0.514	1143	663430 (430478 to 981447)	758587619 (492222327 to 1122218759)
Chile	2472	0.218	484	278377 (179009 to 416043)	134803805 (86685252 to 201468566)
Colombia	1214	0.107	238	743466 (489227 to 1090746)	176807614 (116345671 to 259396156)
Costa Rica	1798	0.158	352	58621 (38012 to 88081)	20647387 (13388454 to 31023503)
Cuba	2863	0.252	561	134334 (92427 to 189978)	75340679 (51837355 to 106547907)
Dominica	676	0.060	132	1022 (635 to 1578)	135399 (84059 to 208902)
Dominican Republic	1124	0.099	220	132979 (85596 to 198722)	29280021 (18847009 to 43755562)
Ecuador	963	0.085	189	243103 (168939 to 343638)	45860324 (31869718 to 64825894)
El Salvador	707	0.062	138	88082 (57070 to 130966)	12199139 (7904062 to 18138360)
Grenada	863	0.076	169	1498 (948 to 2296)	253287 (160292 to 388130)
Guatemala	601	0.053	118	252114 (163320 to 376445)	29681997 (19228012 to 44319729)
Guyana	648	0.057	127	8009 (5079 to 12185)	1016698 (644743 to 1546803)

Haiti	111	0.010	22	156731 (96476 to 250200)	3408004 (2097788 to 5440404)
Honduras	429	0.038	84	128695 (82381 to 193186)	10815367 (6923166 to 16235094)
Jamaica	715	0.063	140	42183 (26690 to 64268)	5908333 (3738289 to 9001647)
Mexico	1171	0.103	229	1580807 (1020792 to 2344997)	362624197 (234161385 to 537923195)
Nicaragua	501	0.044	98	98357 (63177 to 146638)	9653034 (6200397 to 14391510)
Panama	2353	0.207	461	61182 (39093 to 92604)	28201314 (18019568 to 42684615)
Paraguay	968	0.085	190	136436 (88084 to 204165)	25871656 (16702994 to 38714835)
Peru	683	0.060	134	506972 (329731 to 756265)	67830550 (44116507 to 101184929)
Saint Kitts and Nevis	1415	0.125	277	760 (477 to 1160)	210716 (132212 to 321510)
Saint Lucia	744	0.066	146	2816 (1756 to 4353)	410472 (255987 to 634402)
Saint Vincent and the Grenadines	626	0.055	123	1369 (866 to 2098)	167910 (106170 to 257248)
Suriname	1420	0.125	278	6251 (3978 to 9420)	1738918 (1106473 to 2620314)
Trinidad and Tobago	1836	0.162	360	17654 (11240 to 26431)	6349516 (4042740 to 9506081)
United States of America	11345	1.000	2222	8468703 (6335259 to 11116087)	18820991950 (14079587930 to 24704584030)
Uruguay	2156	0.190	422	58053 (37174 to 86115)	24518449 (15700229 to 36370578)
Venezuela (Bolivarian Republic of)	230	0.020	45	337568 (216491 to 505493)	15209350 (9754118 to 22775305)
Eastern Mediterrane-an	832	0.073	163	8359954 (5192932 to 12933545)	1229973714 (781825208 to 1862221123)
Afghanistan	223	0.020	44	435522 (267634 to 675393)	19025508 (11691430 to 29504105)
Bahrain	2260	0.199	443	20990 (13508 to 31386)	9292483 (5980236 to 13895044)

Djibouti	88	0.008	17	15458 (9396 to 24187)	266476 (161966 to 416956)
Egypt	617	0.054	121	1276463 (811655 to 1941197)	154281702 (98101915 to 234625907)
Iran (Islamic Republic of)	1112	0.098	218	1214740 (787147 to 1814987)	264611702 (171467490 to 395366118)
Iraq	415	0.037	81	304370 (194038 to 457367)	24744095 (15774501 to 37182149)
Jordan	734	0.065	144	75682 (47287 to 116263)	10882009 (6799212 to 16717041)
Kuwait	2724	0.240	534	65572 (42015 to 98088)	34990048 (22419867 to 52341293)
Lebanon	1663	0.147	326	45737 (28982 to 69386)	14899732 (9441436 to 22604120)
Libya	1155	0.102	226	66348 (40736 to 103383)	15011778 (9216901 to 23391094)
Morocco	429	0.038	84	702333 (437104 to 1079492)	59023012 (36733566 to 90718936)
Oman	1272	0.112	249	55510 (34670 to 85466)	13831859 (8638997 to 21296259)
Pakistan	158	0.014	31	2310281 (1366561 to 3719840)	71506082 (42296782 to 115133681)
Qatar	3017	0.266	591	53058 (34581 to 77933)	31358191 (20437857 to 46059056)
Saudi Arabia	3223	0.284	631	489834 (313085 to 731605)	309264859 (197671237 to 461910893)
Sudan	252	0.022	49	465491 (286952 to 730111)	22979074 (14165491 to 36042132)
Syrian Arab Republic	1046	0.092	205	145478 (89840 to 227030)	29809112 (18408607 to 46519628)
Tunisia	884	0.078	173	147811 (91909 to 232063)	25596470 (15915914 to 40186366)

United Arab Emirates	3203	0.282	627	179520 (116031 to 269468)	112639552 (72803501 to 169076991)
Yemen	105	0.009	21	289757 (179801 to 448900)	5959971 (3698300 to 9233355)
Europe	3443	0.303	674	19032080 (12364480 to 28231025)	11950286402 (7831077685 to 17572765986)
Albania	816	0.072	160	106587 (68372 to 159680)	17037915 (10929252 to 25524719)
Andorra	3141	0.277	615	1669 (1052 to 2512)	1026916 (646993 to 1545759)
Armenia	1387	0.122	272	87325 (55310 to 131494)	23726504 (15027959 to 35727651)
Austria	6132	0.541	1201	167160 (107421 to 249425)	200795748 (129037056 to 299615274)
Azerbaijan	550	0.048	108	225593 (142796 to 342473)	24305737 (15385130 to 36898671)
Belarus	1163	0.103	228	193389 (124325 to 290585)	44058788 (28324301 to 66202341)
Belgium	5698	0.502	1116	185220 (119077 to 275713)	206743028 (132914301 to 307751791)
Bosnia and Herzegovina	1416	0.125	277	67165 (42087 to 103717)	18630510 (11674381 to 28769757)
Bulgaria	1896	0.167	371	165456 (107330 to 244402)	61452746 (39863884 to 90774497)
Croatia	2020	0.178	396	104535 (66366 to 158577)	41365303 (26261276 to 62749961)
Cyprus	1881	0.166	368	26065 (17012 to 38189)	9604228 (6268608 to 14071816)
Czechia	3296	0.291	646	250137 (163968 to 369404)	161505223 (105868879 to 238512021)
Denmark	6112	0.539	1197	144926 (94074 to 214473)	173520969 (112635339 to 256788933)
Estonia	2623	0.231	514	25948 (17026 to 38313)	13333093 (8748555 to 19686217)

Finland	4680	0.413	917	110513 (73031 to 161701)	101316777 (66953278 to 148245155)
France	5605	0.494	1098	1244801 (808453 to 1838044)	1366773968 (887669783 to 2018146664)
Georgia	1133	0.100	222	87624 (54093 to 136632)	19448024 (12005870 to 30325278)
Germany	6482	0.571	1270	1933070 (1296453 to 2793594)	2454582799 (1646216507 to 3547263406)
Greece	2551	0.225	500	233909 (146529 to 360765)	116890272 (73224086 to 180283604)
Hungary	2321	0.205	455	244272 (160294 to 360771)	111063087 (72880760 to 164031776)
Iceland	5227	0.461	1024	8951 (5913 to 13002)	9165703 (6054351 to 13313240)
Ireland	6362	0.561	1246	98665 (65156 to 143960)	122964467 (81202661 to 179413568)
Israel	3056	0.269	599	141817 (92305 to 209179)	84899065 (55258431 to 125225595)
Italy	3916	0.345	767	1101525 (716805 to 1629673)	845002676 (549875809 to 1250155894)
Kazakhstan	855	0.075	167	316555 (200424 to 480603)	53019587 (33568908 to 80495957)
Kyrgyzstan	350	0.031	69	111394 (69885 to 169156)	7637461 (4791500 to 11597824)
Latvia	2008	0.177	393	40812 (26470 to 60395)	16053711 (10412062 to 23756595)
Lithuania	2562	0.226	502	64060 (41526 to 95813)	32150659 (20840895 to 48086596)
Luxembourg	6436	0.567	1261	9693 (6258 to 14386)	12220409 (7890424 to 18138082)
Malta	4803	0.423	941	9236 (6120 to 13493)	8690159 (5758359 to 12695542)
Monaco	3479	0.307	682	681 (437 to 1012)	463968 (297693 to 689421)
Montenegro	1840	0.162	360	12550 (8018 to 19002)	4523590 (2889973 to 6849291)

Netherlands	6217	0.548	1218	309941 (200687 to 454103)	377468884 (244411779 to 553039185)
North Macedonia	1137	0.100	223	36193 (23421 to 53948)	8061383 (5216653 to 12015877)
Norway	7013	0.618	1374	105477 (69143 to 153727)	144904639 (94989335 to 211190501)
Poland	2256	0.199	442	1050868 (690028 to 1550442)	464417245 (304948676 to 685197437)
Portugal	3350	0.295	656	301813 (199746 to 441038)	198063503 (131082229 to 289428784)
Republic of Moldova	667	0.059	131	73120 (46153 to 112704)	9553943 (6030363 to 14726064)
Romania	1819	0.160	356	690343 (439898 to 1043237)	245990652 (156749240 to 371737712)
Russian Federation	1546	0.136	303	2797912 (1821295 to 4128248)	847353365 (551583035 to 1250248707)
San Marino	4612	0.407	903	711 (448 to 1081)	642211 (404523 to 976797)
Serbia	1302	0.115	255	261118 (165500 to 395870)	66599249 (42211537 to 100968176)
Slovakia	2373	0.209	465	113759 (73836 to 167967)	52881829 (34323263 to 78080309)
Slovenia	3456	0.305	677	47355 (30482 to 70932)	32059636 (20636904 to 48021612)
Spain	3985	0.351	781	662887 (427163 to 995242)	517474281 (333459445 to 776922926)
Sweden	6149	0.542	1205	200153 (131614 to 292180)	241094728 (158535505 to 351946178)
Switzerland	8521	0.751	1669	225069 (147718 to 328509)	375688553 (246572130 to 548351656)
Tajikistan	251	0.022	49	130311 (80356 to 204516)	6407299 (3951042 to 10055914)
Turkey	1316	0.116	258	1532585 (971841 to 2322722)	395094975 (250537207 to 598789640)

Turkmenistan	1247	0.110	244	78198 (47731 to 122996)	19102230 (11659708 to 30045406)
Ukraine	957	0.084	187	817132 (533670 to 1207187)	153188129 (100047398 to 226312066)
United Kingdom	4960	0.437	972	1420350 (947702 to 2052059)	1380060194 (920819263 to 1993850935)
Uzbekistan	391	0.034	77	655482 (411664 to 1012177)	50206384 (31531187 to 77527202)
Southeast Asia	311	0.027	61	29211205 (18763165 to 43939426)	1772139661 (1140611467 to 2658202418)
Bangladesh	129	0.011	25	2631646 (1648882 to 4058204)	66502547 (41667771 to 102552126)
Bhutan	315	0.028	62	20478 (13229 to 30395)	1263646 (816331 to 1875582)
India	253	0.022	50	14999089 (9612079 to 22605378)	743372537 (476385970 to 1120349129)
Indonesia	373	0.033	73	6838649 (4476968 to 10157877)	499689516 (327125143 to 742220386)
Maldives	2145	0.189	420	7333 (4620 to 11238)	3081167 (1941172 to 4722078)
Myanmar	233	0.021	46	1083387 (676400 to 1672536)	49449377 (30873119 to 76340050)
Nepal	188	0.017	37	1002592 (649891 to 1472264)	36923535 (23934238 to 54220643)
Sri Lanka	555	0.049	109	422933 (271615 to 639534)	45981774 (29530337 to 69530873)
Thailand	759	0.067	149	2186076 (1397708 to 3262552)	325033460 (207816163 to 485087824)
Timor-Leste	226	0.020	44	19021 (11773 to 29448)	842102 (521223 to 1303726)
Western Pacific	1670	0.147	327	34383765 (21858117 to 51656303)	9367070134 (6018547464 to 13940183016)
Australia	5398	0.476	1057	456506 (295458 to 672270)	482725782 (312427508 to 710882225)

Brunei Darussalam	1454	0.128	285	5803 (3713 to 8657)	1652817 (1057537 to 2465910)
Cambodia	233	0.021	46	506949 (331633 to 747877)	23138806 (15136820 to 34135571)
China	893	0.079	175	23288737 (14602871 to 35311453)	4073976185 (2554528700 to 6177149820)
Cook Islands	1056	0.093	207	374 (241 to 560)	77381 (49774 to 115762)
Fiji	498	0.044	98	9440 (6091 to 14028)	920898 (594254 to 1368538)
Japan	4787	0.422	938	3471964 (2288493 to 5056641)	3255814668 (2146021994 to 4741837498)
Kiribati	259	0.023	51	1246 (769 to 1954)	63238 (39008 to 99140)
Lao People's Democratic Republic	202	0.018	40	199586 (128025 to 296445)	7897736 (5066033 to 11730521)
Malaysia	1200	0.106	235	493414 (316476 to 742943)	115988160 (74394883 to 174645590)
Marshall Islands	499	0.044	98	530 (330 to 819)	51852 (32283 to 80055)
Micronesia (the Federated States of)	131	0.012	26	1410 (887 to 2148)	36174 (22771 to 55128)
Mongolia	623	0.055	122	54201 (33794 to 83423)	6614840 (4124329 to 10181134)
Nauru	1387	0.122	272	128 (81 to 195)	34715 (22051 to 52922)
New Zealand	4434	0.391	869	99349 (63917 to 149410)	86293892 (55517687 to 129776291)
Niue	849	0.075	166	28 (18 to 42)	4658 (2934 to 7065)
Palau	1817	0.160	356	354 (225 to 532)	125826 (80166 to 189426)
Papua New Guinea	106	0.009	21	138751 (85971 to 216336)	2881137 (1785169 to 4492175)
Philippines	396	0.035	78	1962867 (1301579 to 2858820)	152267513 (100968699 to 221770126)
Republic of Korea	3529	0.311	691	1234289 (795398 to 1833280)	853276135 (549866258 to 1267364376)

Samoa	319	0.028	62	3013 (1882 to 4657)	188304 (117620 to 290995)
Singapore	4645	0.409	910	72210 (45989 to 108084)	65705889 (41846843 to 98348573)
Solomon Islands	116	0.010	23	11144 (7024 to 17178)	253233 (159618 to 390353)
Tonga	290	0.026	57	1423 (888 to 2182)	80816 (50440 to 123954)
Tuvalu	835	0.074	164	182 (114 to 275)	29739 (18716 to 45033)
Vanuatu	92	0.008	18	8783 (5711 to 13057)	158283 (102930 to 235314)
Viet Nam	512	0.045	100	2361085 (1540538 to 3513036)	236811458 (154512437 to 352349522)

Abbreviation: $PPP, 2020 purchasing-power parity-adjusted US$.

Values in parentheses represent uncertainty intervals derived from the sensitivity analyses.

Supplementary Table S10. Health care costs, productivity losses, and total costs of low back pain attributable to occupational ergonomic factors by location in 2019

	Health care costs		Productivity losses		Total costs
Location	Amount, US$	% of total	Amount, US$	% of total	Amount, US$
Global	46982354312 (32326654377 to 66462804489)	21.7	169153744535 (100910248292 to 261824520288)	78.3	216136098847 (133236902669 to 328287324777)
Africa	581160977 (371499053 to 872957890)	14.8	3346876660 (1909427371 to 5357367381)	85.2	3928037636 (2280926423 to 6230325271)
Algeria	62554415 (39245371 to 96418510)	18.3	279566947 (157405943 to 449316903)	81.7	342121362 (196651315 to 545735413)
Angola	13323399 (8255074 to 20632038)	11.5	102031230 (56598491 to 166869356)	88.5	115354629 (64853565 to 187501393)
Benin	3227505 (2051199 to 4919489)	9.6	30447151 (17196822 to 49152621)	90.4	33674656 (19248021 to 54072110)
Botswana	6410946 (4008263 to 9885909)	24.2	20100919 (11320262 to 32558311)	75.8	26511865 (15328525 to 42444219)
Burkina Faso	8447740 (5347992 to 12716563)	20.5	32796191 (18441627 to 52953546)	79.5	41243931 (23789619 to 65670109)
Burundi	3726806 (2412882 to 5529638)	37.2	6288810 (3631520 to 9996337)	62.8	10015616 (6044402 to 15525975)
Cabo Verde	689329 (427511 to 1071197)	18.3	3080639 (1700270 to 5020098)	81.7	3769968 (2127782 to 6091295)
Cameroon	14032913 (9088802 to 20843079)	15.3	77868600 (44086354 to 124977264)	84.7	91901512 (53175156 to 145820344)
Central African Republic	938430 (577461 to 1455664)	14.9	5363419 (2887377 to 8881081)	85.1	6301849 (3464838 to 10336746)
Chad	3647090 (2295507 to 5458716)	13.4	23606693 (12965262 to 38449034)	86.6	27253783 (15260769 to 43907750)
Comoros	593793 (375626 to 899052)	19.5	2446953 (1365259 to 3972262)	80.5	3040746 (1740885 to 4871314)
Congo	970996 (604669 to 1501526)	6.5	13883826 (7636998 to 22888973)	93.5	14854822 (8241667 to 24390499)
Democratic Republic of the Congo	10561576 (6566836 to 16280075)	12.5	74151232 (41520766 to 120891794)	87.5	84712807 (48087603 to 137171869)

Equatorial Guinea	1315448 (812022 to 2054888)	12.0	9655463 (5293763 to 15885764)	88.0	10970910 (6105786 to 17940652)
Eritrea	665457 (420333 to 993937)	18.7	2888150 (1631001 to 4666567)	81.3	3553607 (2051335 to 5660504)
Eswatini	911958 (567037 to 1408397)	22.4	3153042 (1795268 to 5107817)	77.6	4065000 (2362305 to 6516214)
Ethiopia	17160865 (10931141 to 25747933)	18.8	74296567 (42307424 to 119229614)	81.2	91457433 (53238566 to 144977547)
Gabon	1563006 (970225 to 2426746)	16.8	7733561 (4260894 to 12659974)	83.2	9296567 (5231119 to 15086720)
Gambia	619854 (384138 to 952169)	21.0	2331655 (1278352 to 3834427)	79.0	2951509 (1662490 to 4786595)
Ghana	17294429 (11330090 to 25466718)	13.6	109712861 (63250636 to 173958131)	86.4	127007289 (74580726 to 199424849)
Guinea	5470158 (3495338 to 8194201)	13.8	34260675 (18850629 to 55972006)	86.2	39730833 (22345967 to 64166207)
Guinea-Bissau	1038037 (646462 to 1576368)	34.5	1969137 (1082364 to 3228299)	65.5	3007173 (1728826 to 4804668)
Ivory Coast	13736967 (8623053 to 21014540)	13.3	89911897 (50158520 to 146862154)	86.7	103648864 (58781574 to 167876694)
Kenya	42156843 (27080234 to 62985375)	20.9	159407358 (91684752 to 253745107)	79.1	201564202 (118764986 to 316730482)
Lesotho	1517914 (957746 to 2337898)	36.7	2618638 (1466303 to 4256075)	63.3	4136552 (2424049 to 6593973)
Liberia	2396147 (1523861 to 3634300)	37.7	3958744 (2244657 to 6362069)	62.3	6354892 (3768518 to 9996369)
Madagascar	7004180 (4548387 to 10359565)	21.7	25295669 (14535703 to 40341689)	78.3	32299849 (19084090 to 50701254)
Malawi	5690382 (3559210 to 8675764)	39.1	8848573 (4960529 to 14350899)	60.9	14538955 (8519740 to 23026664)
Mali	4704766 (3000400 to 7100749)	12.2	33783976 (18887099 to 54860826)	87.8	38488743 (21887500 to 61961575)
Mauritania	2038833 (1253620 to 3153001)	13.5	13049757 (6935841 to 21849828)	86.5	15088589 (8189460 to 25002829)
Mauritius	6181522 (3999015 to 9221831)	23.8	19771588 (11541263 to 31328689)	76.2	25953110 (15540278 to 40550520)

Mozambiq-ue	11298887 (7366602 to 16612521)	25.6	32770898 (18690605 to 52295784)	74.4	44069785 (26057207 to 68908305)
Namibia	6118347 (3841542 to 9453429)	25.4	17955949 (10110120 to 29168633)	74.6	24074296 (13951662 to 38622062)
Niger	5108588 (3293329 to 7563972)	23.7	16486687 (9369899 to 26370083)	76.3	21595275 (12663228 to 33934055)
Nigeria	123353497 (79777497 to 182634997)	7.7	1482099336 (851745459 to 2360120517)	92.3	1605452833 (931522956 to 2542755514)
Rwanda	8957633 (5819406 to 13222950)	36.3	15722553 (8990222 to 25105991)	63.7	24680186 (14809628 to 38328941)
Sao Tome and Principe	72881 (44814 to 114606)	17.7	338342 (170881 to 582107)	82.3	411223 (215695 to 696713)
Senegal	5869557 (3636163 to 8978107)	15.1	33061163 (18167801 to 54281173)	84.9	38930720 (21803964 to 63259281)
Seychelles	535922 (343546 to 810643)	15.5	2915375 (1670770 to 4648111)	84.5	3451298 (2014316 to 5458754)
Sierra Leone	6253513 (3972762 to 9369040)	41.7	8746764 (4944534 to 14150784)	58.3	15000276 (8917296 to 23519825)
South Africa	86923160 (55570868 to 130745398)	26.2	244652493 (142867683 to 386015676)	73.8	331575654 (198438551 to 516761074)
South Sudan	2530424 (1610783 to 3822302)	27.9	6544992 (3666872 to 10557645)	72.1	9075417 (5277655 to 14379947)
Togo	2464240 (1559001 to 3783762)	16.4	12557700 (7057958 to 20507854)	83.6	15021940 (8616959 to 24291616)
Uganda	18018445 (11607949 to 26663675)	21.9	64330288 (36274767 to 103392112)	78.1	82348733 (47882716 to 130055787)
United Republic of Tanzania	17864829 (11402641 to 26881231)	23.5	58036811 (33344486 to 92892283)	76.5	75901640 (44747127 to 119773515)
Zambia	12603245 (8115227 to 18748321)	20.4	49249834 (28019824 to 79286224)	79.6	61853078 (36135052 to 98034545)
Zimbabwe	12596103 (8177413 to 18637097)	31.7	27127554 (15413539 to 43564890)	68.3	39723657 (23590952 to 62201987)
The Americas	22081723424 (16183093500 to 29556474057)	25.2	65487463161 (40652616254 to 97997109646)	74.8	87569186582 (56835709756 to 127554000000)

Antigua and Barbuda	277483 (176783 to 421464)	12.1	2006400 (1105328 to 3302096)	87.9	2283882 (1282111 to 3723561)
Argentina	274320205 (176281161 to 411243193)	31.1	607042110 (349428454 to 958344885)	68.9	881362316 (525709615 to 1369588078)
Bahamas	1995173 (1276008 to 2993790)	12.6	13871025 (7684965 to 22670543)	87.4	15866198 (8960973 to 25664333)
Barbados	855643 (548360 to 1290170)	10.1	7603833 (4326540 to 12198144)	89.9	8459476 (4874900 to 13488314)
Belize	405814 (258545 to 608616)	13.8	2534629 (1454100 to 4036741)	86.2	2940442 (1712645 to 4645357)
Bolivia (Plurinational State of)	25535039 (16086690 to 38681597)	23.6	82745834 (46784400 to 133220470)	76.4	108280873 (62871090 to 171902067)
Brazil	1109810364 (706603046 to 1664751259)	23.4	3626472323 (2092911995 to 5775914316)	76.6	4736282688 (2799515041 to 7440665576)
Canada	758587619 (492222327 to 1122218759)	20.4	2956087037 (1706246688 to 4677576809)	79.6	3714674657 (2198469015 to 5799795568)
Chile	134803805 (86685252 to 201468566)	23.6	436745576 (252630228 to 694529015)	76.4	571549381 (339315479 to 895997581)
Colombia	176807614 (116345671 to 259396156)	28.9	436035557 (252306615 to 695825308)	71.1	612843172 (368652286 to 955221463)
Costa Rica	20647387 (13388454 to 31023503)	20.9	77917361 (45034586 to 124048344)	79.1	98564748 (58423040 to 155071847)
Cuba	75340679 (51837355 to 106547907)	44.9	92607118 (53260396 to 145995121)	55.1	167947797 (105097751 to 252543028)
Dominica	135399 (84059 to 208902)	16.9	667726 (359161 to 1109039)	83.1	803124 (443220 to 1317941)
Dominican Republic	29280021 (18847009 to 43755562)	19.7	119366235 (68676824 to 190235375)	80.3	148646257 (87523833 to 233990937)
Ecuador	45860324 (31869718 to 64825894)	23.9	145774788 (87472208 to 223579122)	76.1	191635111 (119341926 to 288405017)
El Salvador	12199139 (7904062 to 18138360)	25.3	35978790 (20639479 to 57424683)	74.7	48177929 (28543542 to 75563042)
Grenada	253287 (160292 to 388130)	12.1	1838605 (1028294 to 2980718)	87.9	2091892 (1188586 to 3368848)
Guatemala	29681997 (19228012 to 44319729)	21.1	110893345 (63608918 to 177270293)	78.9	140575342 (82836930 to 221590022)

Guyana	1016698 (644743 to 1546803)	14.7	5881017 (3299336 to 9500109)	85.3	6897715 (3944078 to 11046912)
Haiti	3408004 (2097788 to 5440404)	15.5	18515981 (9600850 to 31460345)	84.5	21923986 (11698638 to 36900749)
Honduras	10815367 (6923166 to 16235094)	18.6	47205339 (26947306 to 75874768)	81.4	58020706 (33870472 to 92109862)
Jamaica	5908333 (3738289 to 9001647)	17.6	27641881 (15576047 to 44546219)	82.4	33550214 (19314336 to 53547866)
Mexico	362624197 (234161385 to 537923195)	24.6	1109851902 (643609760 to 1750848925)	75.4	1472476099 (877771145 to 2288772120)
Nicaragua	9653034 (6200397 to 14391510)	29.6	23012953 (12908747 to 37505293)	70.4	32665988 (19109144 to 51896804)
Panama	28201314 (18019568 to 42684615)	29.2	68484101 (39065561 to 109951594)	70.8	96685415 (57085129 to 152636210)
Paraguay	25871656 (16702994 to 38714835)	25.4	75948619 (43945091 to 121022611)	74.6	101820275 (60648085 to 159737447)
Peru	67830550 (44116507 to 101184929)	18.8	292741666 (170386211 to 465271205)	81.2	360572216 (214502718 to 566456134)
Saint Kitts and Nevis	210716 (132212 to 321510)	13.7	1326194 (722939 to 2172723)	86.3	1536910 (855151 to 2494233)
Saint Lucia	410472 (255987 to 634402)	13.7	2596246 (1442093 to 4210404)	86.3	3006717 (1698080 to 4844806)
Saint Vincent and the Grenadines	167910 (106170 to 257248)	16.1	874404 (483806 to 1439856)	83.9	1042313 (589976 to 1697104)
Suriname	1738918 (1106473 to 2620314)	31.4	3803411 (2179745 to 6103854)	68.6	5542329 (3286218 to 8724168)
Trinidad and Tobago	6349516 (4042740 to 9506081)	15.4	34852395 (19891927 to 55899786)	84.6	41201911 (23934667 to 65405867)
United States of America	18820991950 (14079587930 to 24704584030)	25.6	54734323887 (34446209172 to 80927333495)	74.4	73555315833 (48525797104 to 105631917524)
Uruguay	24518449 (15700229 to 36370578)	22.8	82948794 (47786919 to 130649449)	77.2	107467243 (63487149 to 167020027)
Venezuela (Bolivarian Republic of)	15209350 (9754118 to 22775305)	7.0	201266078 (113601565 to 323057987)	93.0	216475428 (123355683 to 345833292)

Eastern Mediterran-ean	1229973714 (781825208 to 1862221123)	23.1	4097479417 (2337865450 to 6557026769)	76.9	5327453132 (3119690658 to 8419247892)
Afghanistan	19025508 (11691430 to 29504105)	35.5	34630807 (18914834 to 57421744)	64.5	53656315 (30606264 to 86925849)
Bahrain	9292483 (5980236 to 13895044)	23.0	31138710 (18005369 to 49570071)	77.0	40431193 (23985605 to 63465115)
Djibouti	266476 (161966 to 416956)	5.9	4224600 (2201581 to 7123915)	94.1	4491076 (2363547 to 7540871)
Egypt	154281702 (98101915 to 234625907)	25.3	454481472 (256065937 to 732131684)	74.7	608763174 (354167853 to 966757591)
Iran (Islamic Republic of)	264611702 (171467490 to 395366118)	26.2	747199075 (434880770 to 1184737054)	73.8	1011810777 (606348261 to 1580103172)
Iraq	24744095 (15774501 to 37182149)	13.9	153416556 (87360954 to 243998993)	86.1	178160651 (103135455 to 281181142)
Jordan	10882009 (6799212 to 16717041)	20.2	42922414 (24772196 to 68705033)	79.8	53804423 (31571409 to 85422074)
Kuwait	34990048 (22419867 to 52341293)	22.3	121832717 (70023967 to 193752713)	77.7	156822765 (92443834 to 246094006)
Lebanon	14899732 (9441436 to 22604120)	25.9	42552023 (23772006 to 69130437)	74.1	57451755 (33213442 to 91734557)
Libya	15011778 (9216901 to 23391094)	18.9	64610361 (34695385 to 107055253)	81.1	79622139 (43912286 to 130446347)
Morocco	59023012 (36733566 to 90718936)	16.2	304262069 (166400496 to 499920448)	83.8	363285081 (203134062 to 590639384)
Oman	13831859 (8638997 to 21296259)	10.0	123933016 (70881769 to 197146170)	90.0	137764875 (79520766 to 218442429)
Pakistan	71506082 (42296782 to 115133681)	18.8	309465604 (165235508 to 520407880)	81.2	380971685 (207532290 to 635541560)
Qatar	31358191 (20437857 to 46059056)	22.5	107756390 (62826709 to 169214695)	77.5	139114581 (83264566 to 215273751)
Saudi Arabia	309264859 (197671237 to 461910893)	24.6	946095356 (552324329 to 1487142234)	75.4	1255360216 (749995566 to 1949053127)

Sudan	22979074 (14165491 to 36042132)	34.0	44587712 (23985625 to 74483216)	66.0	67566786 (38151115 to 110525348)
Syrian Arab Republic	29809112 (18408607 to 46519628)	54.3	25075002 (13829638 to 41711047)	45.7	54884114 (32238245 to 88230675)
Tunisia	25596470 (15915914 to 40186366)	28.7	63726081 (35896119 to 103787135)	71.3	89322551 (51812033 to 143973500)
United Arab Emirates	112639552 (72803501 to 169076991)	20.4	440615523 (256406650 to 692097219)	79.6	553255074 (329210150 to 861174210)
Yemen	5959971 (3698300 to 9233355)	14.6	34953930 (19385607 to 57489828)	85.4	40913901 (23083907 to 66723183)
Europe	11950286402 (7831077685 to 17572765986)	20.0	47761028627 (27963073604 to 74841192638)	80.0	59711315029 (35794151289 to 92413958623)
Albania	17037915 (10929252 to 25524719)	24.4	52815527 (29729789 to 84341323)	75.6	69853442 (40659040 to 109866042)
Andorra	1026916 (646993 to 1545759)	11.7	7721794 (4240007 to 12618486)	88.3	8748710 (4887000 to 14164245)
Armenia	23726504 (15027959 to 35727651)	35.9	42279126 (23781422 to 68190981)	64.1	66005630 (38809381 to 103918632)
Austria	200795748 (129037056 to 299615274)	19.7	816609300 (474664058 to 1288493167)	80.3	1017405048 (603701114 to 1588108441)
Azerbaijan	24305737 (15385130 to 36898671)	19.6	99972594 (56758279 to 160614966)	80.4	124278331 (72143409 to 197513637)
Belarus	44058788 (28324301 to 66202341)	28.2	112453893 (63886496 to 179592787)	71.8	156512680 (92210797 to 245795128)
Belgium	206743028 (132914301 to 307751791)	16.6	1039411788 (603889999 to 1636861540)	83.4	1246154816 (736804300 to 1944613331)
Bosnia and Herzegovina	18630510 (11674381 to 28769757)	37.8	30636573 (17519822 to 49285827)	62.2	49267083 (29194202 to 78055584)
Bulgaria	61452746 (39863884 to 90774497)	31.3	135026650 (79213789 to 210673370)	68.7	196479396 (119077673 to 301447866)
Croatia	41365303 (26261276 to 62749961)	19.6	169249807 (97298514 to 268899570)	80.4	210615110 (123559790 to 331649532)

Cyprus	9604228 (6268608 to 14071816)	15.4	52679903 (30723538 to 82580900)	84.6	62284132 (36992145 to 96652716)
Czechia	161505223 (105868879 to 238512021)	22.2	564402634 (333393661 to 880083293)	77.8	725907856 (439262541 to 1118595314)
Denmark	173520969 (112635339 to 256788933)	16.7	865607730 (500778787 to 1365633988)	83.3	1039128698 (613414127 to 1622422921)
Estonia	13333093 (8748555 to 19686217)	19.5	54932147 (32049365 to 86474380)	80.5	68265240 (40797920 to 106160597)
Finland	101316777 (66953278 to 148245155)	16.5	512971314 (298766603 to 808819841)	83.5	614288092 (365719882 to 957064995)
France	1366773968 (887669783 to 2018146664)	19.3	5708313071 (3333435964 to 8945335568)	80.7	7075087038 (4221105748 to 10963482232)
Georgia	19448024 (12005870 to 30325278)	38.4	31145336 (17501257 to 50538514)	61.6	50593360 (29507127 to 80863792)
Germany	2454582799 (1646216507 to 3547263406)	21.3	9093431857 (5395171849 to 14110638165)	78.7	11548014656 (7041388356 to 17657901570)
Greece	116890272 (73224086 to 180283604)	22.3	407361956 (228993502 to 654132058)	77.7	524252229 (302217588 to 834415662)
Hungary	111063087 (72880760 to 164031776)	22.3	386689774 (227923350 to 599608351)	77.7	497752861 (300804110 to 763640127)
Iceland	9165703 (6054351 to 13313240)	12.4	64465411 (37547826 to 101675421)	87.6	73631113 (43602177 to 114988661)
Ireland	122964467 (81202661 to 179413568)	14.9	701712020 (409727651 to 1099410497)	85.1	824676487 (490930313 to 1278824065)
Israel	84899065 (55258431 to 125225595)	11.4	661181643 (378770918 to 1048383182)	88.6	746080709 (434029349 to 1173608777)
Italy	845002676 (549875809 to 1250155894)	17.5	3975232305 (2333736672 to 6191070376)	82.5	4820234982 (2883612481 to 7441226270)
Kazakhstan	53019587 (33568908 to 80495957)	14.9	303956752 (174660041 to 486241836)	85.1	356976339 (208228949 to 566737794)
Kyrgyzstan	7637461 (4791500 to 11597824)	31.6	16526148 (9292080 to 26706277)	68.4	24163609 (14083580 to 38304101)

Latvia	16053711 (10412062 to 23756595)	21.3	59404045 (34771942 to 93698278)	78.7	75457756 (45184004 to 117454873)
Lithuania	32150659 (20840895 to 48086596)	25.0	96530882 (56038573 to 151818974)	75.0	128681542 (76879468 to 199905570)
Luxembourg	12220409 (7890424 to 18138082)	9.7	113827231 (65954306 to 178628683)	90.3	126047640 (73844730 to 196766765)
Malta	8690159 (5758359 to 12695542)	27.4	23082717 (13490754 to 36050875)	72.6	31772876 (19249113 to 48746416)
Monaco	463968 (297693 to 689421)	3.7	12090305 (6930834 to 19148635)	96.3	12554274 (7228527 to 19838056)
Montenegro	4523590 (2889973 to 6849291)	33.6	8946117 (5128426 to 14262096)	66.4	13469706 (8018399 to 21111387)
Netherlands	377468884 (244411779 to 553039185)	18.5	1665911846 (959815043 to 2639210011)	81.5	2043380730 (1204226822 to 3192249196)
North Macedonia	8061383 (5216653 to 12015877)	30.8	18085375 (10579954 to 28394580)	69.2	26146758 (15796607 to 40410457)
Norway	144904639 (94989335 to 211190501)	17.9	665728946 (386515719 to 1040768578)	82.1	810633586 (481505053 to 1251959079)
Poland	464417245 (304948676 to 685197437)	24.2	1451054110 (850346706 to 2262447351)	75.8	1915471355 (1155295382 to 2947644788)
Portugal	198063503 (131082229 to 289428784)	22.2	692730779 (408981879 to 1083740593)	77.8	890794282 (540064109 to 1373169377)
Republic of Moldova	9553943 (6030363 to 14726064)	17.9	43952031 (25022630 to 70786240)	82.1	53505974 (31052993 to 85512304)
Romania	245990652 (156749240 to 371737712)	25.8	706105247 (405193226 to 1123692874)	74.2	952095899 (561942466 to 1495430587)
Russian Federation	847353365 (551583035 to 1250248707)	22.2	2977272033 (1758383338 to 4672656944)	77.8	3824625398 (2309966373 to 5922905651)
San Marino	642211 (404523 to 976797)	21.3	2367905 (1375875 to 3722641)	78.7	3010116 (1780398 to 4699438)
Serbia	66599249 (42211537 to 100968176)	26.5	184567491 (105296163 to 295062730)	73.5	251166739 (147507700 to 396030906)

Slovakia	52881829 (34323263 to 78080309)	21.4	194223171 (113395735 to 303222883)	78.6	247105000 (147718998 to 381303192)
Slovenia	32059636 (20636904 to 48021612)	19.7	130604355 (76131404 to 205926465)	80.3	162663991 (96768308 to 253948077)
Spain	517474281 (333459445 to 776922926)	19.6	2125962300 (1237522065 to 3377426173)	80.4	2643436581 (1570981510 to 4154349099)
Sweden	241094728 (158535505 to 351946178)	20.1	956738418 (558864398 to 1494524459)	79.9	1197833146 (717399903 to 1846470638)
Switzerland	375688553 (246572130 to 548351656)	15.0	2136088392 (1244022631 to 3343509731)	85.0	2511776945 (1490594761 to 3891861388)
Tajikistan	6407299 (3951042 to 10055914)	33.9	12490485 (6533837 to 21262035)	66.1	18897784 (10484879 to 31317949)
Turkey	395094975 (250537207 to 598789640)	27.3	1049558194 (596840849 to 1696193883)	72.7	1444653169 (847378056 to 2294983524)
Turkmenist-an	19102230 (11659708 to 30045406)	20.9	72304321 (38298773 to 121181252)	79.1	91406552 (49958482 to 151226658)
Ukraine	153188129 (100047398 to 226312066)	31.9	327306256 (189898701 to 515552889)	68.1	480494385 (289946098 to 741864955)
United Kingdom	1380060194 (920819263 to 1993850935)	18.7	6011681861 (3549357933 to 9364119777)	81.3	7391742055 (4470177197 to 11357970713)
Uzbekistan	50206384 (31531187 to 77527202)	30.3	115626758 (64926670 to 187278345)	69.7	165833143 (96457857 to 264805547)
Southeast Asia	1772139661 (1140611467 to 2658202418)	17.0	8633088708 (4984457469 to 13758060977)	83.0	10405228369 (6125068936 to 16416263394)
Bangladesh	66502547 (41667771 to 102552126)	12.2	479570527 (267301111 to 783148801)	87.8	546073074 (308968882 to 885700927)
Bhutan	1263646 (816331 to 1875582)	17.2	6062838 (3441542 to 9715474)	82.8	7326483 (4257873 to 11591055)
India	743372537 (476385970 to 1120349129)	15.5	4047510561 (2349415056 to 6432421867)	84.5	4790883099 (2825801025 to 7552770996)
Indonesia	499689516 (327125143 to 742220386)	18.2	2239549380 (1304123968 to 3538648990)	81.8	2739238895 (1631249111 to 4280869377)

Maldives	3081167 (1941172 to 4722078)	31.2	6799939 (3854078 to 10920274)	68.8	9881106 (5795251 to 15642352)
Myanmar	49449377 (30873119 to 76340050)	25.7	142844106 (80252701 to 230732592)	74.3	192293483 (111125820 to 307072643)
Nepal	36923535 (23934238 to 54220643)	33.6	73116654 (41958698 to 116337801)	66.4	110040189 (65892936 to 170558445)
Sri Lanka	45981774 (29530337 to 69530873)	25.4	135009501 (77212944 to 217229779)	74.6	180991275 (106743281 to 286760653)
Thailand	325033460 (207816163 to 485087824)	17.8	1500075849 (855518161 to 2414640288)	82.2	1825109309 (1063334324 to 2899728112)
Timor-Leste	842102 (521223 to 1303726)	24.8	2549354 (1379209 to 4265108)	75.2	3391456 (1900432 to 5568834)
Western Pacific	9367070134 (6018547464 to 13940183016)	19.0	39827807965 (23062808144 to 63313762879)	81.0	49194878098 (29081355608 to 77253945894)
Australia	482725782 (312427508 to 710882225)	16.8	2396700055 (1382397585 to 3787409670)	83.2	2879425837 (1694825093 to 4498291895)
Brunei Darussalam	1652817 (1057537 to 2465910)	8.4	18044650 (10225204 to 28559638)	91.6	19697467 (11282741 to 31025547)
Cambodia	23138806 (15136820 to 34135571)	26.6	63702166 (36488369 to 100934372)	73.4	86840971 (51625189 to 135069943)
China	4073976185 (2554528700 to 6177149820)	17.2	19588599793 (11211783304 to 31541223187)	82.8	23662575977 (13766312003 to 37718373008)
Cook Islands	77381 (49774 to 115762)	9.2	761412 (440721 to 1214899)	90.8	838792 (490495 to 1330661)
Fiji	920898 (594254 to 1368538)	12.2	6639859 (3708279 to 10736176)	87.8	7560757 (4302533 to 12104713)
Japan	3255814668 (2146021994 to 4741837498)	21.8	11687823814 (6890272951 to 18240743165)	78.2	14943638481 (9036294944 to 22982580661)
Kiribati	63238 (39008 to 99140)	11.6	480159 (254681 to 802770)	88.4	543397 (293690 to 901910)
Lao People's Democratic Republic	7897736 (5066033 to 11730521)	12.6	54973601 (31064286 to 88102668)	87.4	62871338 (36130319 to 99833189)
Malaysia	115988160 (74394883 to 174645590)	21.2	432351071 (248518725 to 690766238)	78.8	548339231 (322913608 to 865411827)
Marshall Islands	51852 (32283 to 80055)	12.7	354853 (187616 to 598830)	87.3	406705 (219900 to 678886)

Micronesia (the Federated States of)	36174 (22771 to 55128)	5.9	579155 (326618 to 934076)	94.1	615329 (349390 to 989204)
Mongolia	6614840 (4124329 to 10181134)	22.8	22391706 (12651275 to 35954742)	77.2	29006547 (16775604 to 46135876)
Nauru	34715 (22051 to 52922)	17.1	168315 (94698 to 273209)	82.9	203030 (116749 to 326131)
New Zealand	86293892 (55517687 to 129776291)	20.1	343866343 (197476367 to 547606977)	79.9	430160235 (252994054 to 677383268)
Niue	4658 (2934 to 7065)	12.7	31883 (17185 to 52964)	87.3	36540 (20119 to 60029)
Palau	125826 (80166 to 189426)	20.2	497127 (284809 to 794608)	79.8	622953 (364975 to 984034)
Papua New Guinea	2881137 (1785169 to 4492175)	6.2	43608844 (23441625 to 72818074)	93.8	46489981 (25226794 to 77310249)
Philippines	152267513 (100968699 to 221770126)	23.2	503889213 (296797955 to 788374202)	76.8	656156726 (397766654 to 1010144328)
Republic of Korea	853276135 (549866258 to 1267364376)	19.2	3581590027 (2092048330 to 5662584259)	80.8	4434866162 (2641914588 to 6929948635)
Samoa	188304 (117620 to 290995)	7.8	2220792 (1195206 to 3735438)	92.2	2409096 (1312826 to 4026433)
Singapore	65705889 (41846843 to 98348573)	14.6	384635460 (217931921 to 615066510)	85.4	450341349 (259778764 to 713415084)
Solomon Islands	253233 (159618 to 390353)	8.7	2657419 (1480933 to 4308519)	91.3	2910652 (1640551 to 4698873)
Tonga	80816 (50440 to 123954)	6.9	1086791 (555994 to 1856520)	93.1	1167607 (606434 to 1980474)
Tuvalu	29739 (18716 to 45033)	25.2	88091 (49474 to 142242)	74.8	117830 (68190 to 187274)
Vanuatu	158283 (102930 to 235314)	3.9	3951613 (2217505 to 6347699)	96.1	4109897 (2320436 to 6583013)
Viet Nam	236811458 (154512437 to 352349522)	25.7	686113754 (400896528 to 1081821228)	74.3	922925212 (555408965 to 1434170750)

Values in parentheses represent uncertainty intervals derived from the sensitivity analyses.

Percentage calculations were based on the mean values.

Supplementary Table S11. Health care costs of low back pain attributable to occupational ergonomic factors borne by sector and location in 2019

	The public sector		The private sector/third party		The out-of-pocket sector	
Location	Amount, US$	% of health care costs	Amount, US$	% of health care costs	Amount, US$	% of health care costs
Global	27797951670 (19005472962 to 39516249859)	59.2	10061686300 (7224699785 to 13720577582)	21.4	9104525976 (6082830217 to 13202184477)	19.4
Africa	210641826 (134209573 to 317917566)	36.2	156088529 (99935425 to 233908917)	26.9	214232875 (137227579 to 320834373)	36.9
Algeria	40660370 (25509491 to 62672032)	65.0	938316 (588681 to 1446278)	1.5	20893175 (13107954 to 32203782)	33.4
Angola	5489240 (3401091 to 8500400)	41.2	2837884 (1758331 to 4394624)	21.3	4996275 (3095653 to 7737014)	37.5
Benin	732644 (465622 to 1116724)	22.7	977934 (621513 to 1490605)	30.3	1516927 (964063 to 2312160)	47.0
Botswana	5032592 (3146487 to 7760438)	78.5	1173203 (733512 to 1809121)	18.3	198739 (124256 to 306463)	3.1
Burkina Faso	3531155 (2235461 to 5315523)	41.8	1985219 (1256778 to 2988392)	23.5	2931366 (1855753 to 4412647)	34.7
Burundi	1244753 (805903 to 1846899)	33.4	1565258 (1013410 to 2322448)	42.0	920521 (595982 to 1365821)	24.7
Cabo Verde	453579 (281302 to 704848)	65.8	64108 (39759 to 99621)	9.3	171643 (106450 to 266728)	24.9
Cameroon	477119 (309019 to 708665)	3.4	3381932 (2190401 to 5023182)	24.1	10173862 (6589381 to 15111233)	72.5
Central African Republic	99474 (61211 to 154300)	10.6	273083 (168041 to 423598)	29.1	565873 (348209 to 877765)	60.3
Chad	630947 (397123 to 944358)	17.3	926361 (583059 to 1386514)	25.4	2086136 (1313030 to 3122386)	57.2
Comoros	95601 (60476 to 144747)	16.1	131228 (83013 to 198690)	22.1	366964 (232137 to 555614)	61.8
Congo	365094 (227355 to 564574)	37.6	160214 (99770 to 247752)	16.5	445687 (277543 to 689200)	45.9

Democratic Republic of the Congo	1668729 (1037560 to 2572252)	15.8	4826640 (3001044 to 7439994)	45.7	4066207 (2528232 to 6267829)	38.5
Equatorial Guinea	280190 (172961 to 437691)	21.3	43410 (26797 to 67811)	3.3	993163 (613077 to 1551441)	75.5
Eritrea	117121 (73979 to 174933)	17.6	260859 (164771 to 389623)	39.2	287478 (181584 to 429381)	43.2
Eswatini	462363 (287488 to 714057)	50.7	352928 (219443 to 545050)	38.7	96668 (60106 to 149290)	10.6
Ethiopia	3895516 (2481369 to 5844781)	22.7	6761381 (4306870 to 10144686)	39.4	6503968 (4142903 to 9758467)	37.9
Gabon	942493 (585046 to 1463328)	60.3	259459 (161057 to 402840)	16.6	361054 (224122 to 560578)	23.1
Gambia	168600 (104486 to 258990)	27.2	307447 (190532 to 472276)	49.6	143806 (89120 to 220903)	23.2
Ghana	6952360 (4554696 to 10237621)	40.2	4081485 (2673901 to 6010145)	23.6	6260583 (4101493 to 9218952)	36.2
Guinea	1230785 (786451 to 1843695)	22.5	1001039 (639647 to 1499539)	18.3	3238333 (2069240 to 4850967)	59.2
Guinea-Bissau	66434 (41374 to 100888)	6.4	294802 (183595 to 447689)	28.4	676800 (421494 to 1027792)	65.2
Ivory Coast	3997457 (2509309 to 6115231)	29.1	4615621 (2897346 to 7060885)	33.6	5123889 (3216399 to 7838423)	37.3
Kenya	19392148 (12456908 to 28973273)	46.0	12520582 (8042830 to 18706656)	29.7	10244113 (6580497 to 15305446)	24.3
Lesotho	660293 (416620 to 1016986)	43.5	648149 (408958 to 998282)	42.7	209472 (132169 to 322630)	13.8
Liberia	385780 (245342 to 585122)	16.1	706863 (449539 to 1072119)	29.5	1303504 (828980 to 1977059)	54.4
Madagascar	2255346 (1464581 to 3335780)	32.2	2472475 (1605581 to 3656926)	35.3	2276358 (1478226 to 3366859)	32.5
Malawi	1855065 (1160303 to 2828299)	32.6	2879334 (1800960 to 4389937)	50.6	961675 (601507 to 1466204)	16.9
Mali	1580801 (1008135 to 2385852)	33.6	1646668 (1050140 to 2485262)	35.0	1477297 (942126 to 2229635)	31.4
Mauritania	764562 (470107 to 1182376)	37.5	356796 (219383 to 551775)	17.5	917475 (564129 to 1418851)	45.0
Mauritius	2905315 (1879537 to 4334261)	47.0	445070 (287929 to 663972)	7.2	2824956 (1827550 to 4214377)	45.7

Mozambiq-ue	2406663 (1569086 to 3538467)	21.3	7762335 (5060855 to 11412802)	68.7	1129889 (736660 to 1661252)	10.0
Namibia	2869505 (1801683 to 4433658)	46.9	2747138 (1724853 to 4244590)	44.9	501704 (315006 to 775181)	8.2
Niger	1823766 (1175718 to 2700338)	35.7	929763 (599386 to 1376643)	18.2	2355059 (1518225 to 3486991)	46.1
Nigeria	19613206 (12684622 to 29038965)	15.9	16652722 (10769962 to 24655725)	13.5	86964216 (56243135 to 128757673)	70.5
Rwanda	3574096 (2321943 to 5275957)	39.9	4335495 (2816593 to 6399908)	48.4	1048043 (680871 to 1547085)	11.7
Sao Tome and Principe	34473 (21197 to 54209)	47.3	25581 (15730 to 40227)	35.1	12900 (7932 to 20285)	17.7
Senegal	1467389 (909041 to 2244527)	25.0	1402824 (869043 to 2145768)	23.9	2993474 (1854443 to 4578835)	51.0
Seychelles	389616 (249758 to 589337)	72.7	10718 (6871 to 16213)	2.0	135052 (86574 to 204282)	25.2
Sierra Leone	875492 (556187 to 1311666)	14.0	1926082 (1223611 to 2885664)	30.8	3451939 (2192965 to 5171710)	55.2
South Africa	51110818 (32675670 to 76878294)	58.8	30857722 (19727658 to 46414616)	35.5	4954620 (3167539 to 7452488)	5.7
South Sudan	412459 (262558 to 623035)	16.3	1523315 (969691 to 2301026)	60.2	594650 (378534 to 898241)	23.5
Togo	372100 (235409 to 571348)	15.1	460813 (291533 to 707563)	18.7	1631327 (1032059 to 2504850)	66.2
Uganda	2720785 (1752800 to 4026215)	15.1	8396595 (5409304 to 12425273)	46.6	6901064 (4445845 to 10212188)	38.3
United Republic of Tanzania	7306715 (4663680 to 10994424)	40.9	6592122 (4207575 to 9919174)	36.9	3965992 (2531386 to 5967633)	22.2
Zambia	5053901 (3254206 to 7518077)	40.1	6263813 (4033268 to 9317916)	49.7	1285531 (827753 to 1912329)	10.2
Zimbabwe	2216914 (1439225 to 3280129)	17.6	7305740 (4742900 to 10809516)	58.0	3073449 (1995289 to 4547452)	24.4

The Americas	11370166454 (8322844622 to 15236454114)	51.5	7750263986 (5729819493 to 10288204658)	35.1	2941752780 (2115896779 to 4006025981)	13.3
Antigua and Barbuda	162050 (103241 to 246135)	58.4	47727 (30407 to 72492)	17.2	67428 (42958 to 102416)	24.3
Argentina	171175808 (109999445 to 256615752)	62.4	27432021 (17628116 to 41124319)	10.0	75986697 (48829882 to 113914364)	27.7
Bahamas	1037490 (663524 to 1556771)	52.0	434948 (278170 to 652646)	21.8	522735 (334314 to 784373)	26.2
Barbados	383328 (245665 to 577996)	44.8	72730 (46611 to 109664)	8.5	399585 (256084 to 602509)	46.7
Belize	283664 (180723 to 425423)	69.9	33683 (21459 to 50515)	8.3	88467 (56363 to 132678)	21.8
Bolivia (Plurinational State of)	18180948 (11453723 to 27541297)	71.2	1251217 (788248 to 1895398)	4.9	6102874 (3844719 to 9244902)	23.9
Brazil	451692818 (287587440 to 677553763)	40.7	380664955 (242364845 to 571009682)	34.3	276342781 (175944158 to 414523064)	24.9
Canada	532528509 (345540074 to 787797569)	70.2	113029555 (73341127 to 167210595)	14.9	113029555 (73341127 to 167210595)	14.9
Chile	68615137 (44122793 to 102547500)	50.9	21973020 (14129696 to 32839376)	16.3	44215648 (28432763 to 66081690)	32.8
Colombia	127124675 (83652537 to 186505836)	71.9	23515413 (15473974 to 34499689)	13.3	26344335 (17335505 to 38650027)	14.9
Costa Rica	14969355 (9706629 to 22492040)	72.5	1053017 (682811 to 1582199)	5.1	4604367 (2985625 to 6918241)	22.3
Cuba	67279226 (46290758 to 95147281)	89.3	75341 (51837 to 106548)	0.1	7986112 (5494760 to 11294078)	10.6
Dominica	87332 (54218 to 134742)	64.5	2302 (1429 to 3551)	1.7	45900 (28496 to 70818)	33.9
Dominican Republic	13146730 (8462307 to 19646247)	44.9	3542883 (2280488 to 5294423)	12.1	12561129 (8085367 to 18771136)	42.9
Ecuador	28341680 (19695486 to 40062403)	61.8	3347804 (2326489 to 4732290)	7.3	14170840 (9847743 to 20031201)	30.9
El Salvador	7746453 (5019080 to 11517858)	63.5	988130 (640229 to 1469207)	8.1	3464555 (2244754 to 5151294)	28.4
Grenada	104861 (66361 to 160686)	41.4	10638 (6732 to 16301)	4.2	137788 (87199 to 211143)	54.4

Guatemala	11397887 (7383557 to 17018776)	38.4	1662192 (1076769 to 2481905)	5.6	16621918 (10767687 to 24819048)	56.0
Guyana	603918 (382977 to 918801)	59.4	57952 (36750 to 88168)	5.7	353811 (224370 to 538288)	34.8
Haiti	374880 (230757 to 598444)	11.0	1557458 (958689 to 2486265)	45.7	1475666 (908342 to 2355695)	43.3
Honduras	4239624 (2713881 to 6364157)	39.2	886860 (567700 to 1331278)	8.2	5688883 (3641585 to 8539660)	52.6
Jamaica	3858141 (2441103 to 5878076)	65.3	1081225 (684107 to 1647301)	18.3	968967 (613079 to 1476270)	16.4
Mexico	178773729 (115441563 to 265196135)	49.3	31185681 (20137879 to 46261395)	8.6	152664787 (98581943 to 226465665)	42.1
Nicaragua	5878698 (3776042 to 8764430)	60.9	444040 (285218 to 662009)	4.6	3320644 (2132937 to 4950680)	34.4
Panama	18641068 (11910934 to 28214531)	66.1	1776683 (1135233 to 2689131)	6.3	7783563 (4973401 to 11780954)	27.6
Paraguay	11900962 (7683377 to 17808824)	46.0	3208085 (2071171 to 4800640)	12.4	10762609 (6948445 to 16105371)	41.6
Peru	42665416 (27749283 to 63645320)	62.9	6104750 (3970486 to 9106644)	9.0	19060385 (12396739 to 28432965)	28.1
Saint Kitts and Nevis	103883 (65181 to 158505)	49.3	9271 (5817 to 14146)	4.4	97772 (61347 to 149181)	46.4
Saint Lucia	194564 (121338 to 300707)	47.4	25860 (16127 to 39967)	6.3	190048 (118522 to 293728)	46.3
Saint Vincent and the Grenadines	111324 (70391 to 170555)	66.3	7724 (4884 to 11833)	4.6	48862 (30896 to 74859)	29.1
Suriname	1252021 (796661 to 1886626)	72.0	206931 (131670 to 311817)	11.9	279966 (178142 to 421871)	16.1
Trinidad and Tobago	2920777 (1859660 to 4372797)	46.0	450816 (287035 to 674932)	7.1	2977923 (1896045 to 4458352)	46.9
United States of America	9561063910 (7152430668 to 12549928688)	50.8	7114334957 (5322084237 to 9338332764)	37.8	2126772090 (1590993436 to 2791617995)	11.3
Uruguay	16329287 (10456353 to 24222805)	66.6	4388802 (2810341 to 6510333)	17.9	3800360 (2433536 to 5637440)	15.5

Venezuela (Bolivarian Republic of)	6996301 (4486894 to 10476640)	46.0	5399319 (3462712 to 8085233)	35.5	2813730 (1804512 to 4213431)	18.5
Eastern Mediterran-ean	634193946 (404393522 to 956331321)	51.6	164321868 (104761390 to 248202035)	13.4	431487106 (272688321 to 657733459)	35.1
Afghanistan	1560092 (958697 to 2419337)	8.2	2378189 (1461429 to 3688013)	12.5	15087228 (9271304 to 23396755)	79.3
Bahrain	5501150 (3540300 to 8225866)	59.2	1031466 (663806 to 1542350)	11.1	2759867 (1776130 to 4126828)	29.7
Djibouti	143098 (86976 to 223905)	53.7	59158 (35956 to 92564)	22.2	64487 (39196 to 100903)	24.2
Egypt	42890313 (27272332 to 65226002)	27.8	14656762 (9319682 to 22289461)	9.5	96734627 (61509901 to 147110444)	62.7
Iran (Islamic Republic of)	130982793 (84876408 to 195706228)	49.5	29107287 (18861424 to 43490273)	11.0	104521622 (67729659 to 156169617)	39.5
Iraq	12223583 (7792603 to 18367981)	49.4	123720 (78873 to 185911)	0.5	12396792 (7903025 to 18628256)	50.1
Jordan	5571589 (3481197 to 8559125)	51.2	2013172 (1257854 to 3092653)	18.5	3297249 (2060161 to 5065263)	30.3
Kuwait	30441342 (19505284 to 45536925)	87.0	419881 (269038 to 628096)	1.2	4128826 (2645544 to 6176273)	11.8
Lebanon	7300868 (4626304 to 11076019)	49.0	2607453 (1652251 to 3955721)	17.5	4991410 (3162881 to 7572380)	33.5
Libya	9502455 (5834298 to 14806562)	63.3	0 (0 to 0)	0.0	5509323 (3382603 to 8584531)	36.7
Morocco	23550182 (14656693 to 36196855)	39.9	7850061 (4885564 to 12065618)	13.3	27622770 (17191309 to 42456462)	46.8
Oman	11950727 (7464093 to 18399968)	86.4	968230 (604730 to 1490738)	7.0	912903 (570174 to 1405553)	6.6
Pakistan	22881946 (13534970 to 36842778)	32.0	10153864 (6006143 to 16348983)	14.2	38470272 (22755669 to 61941920)	53.8
Qatar	22828763 (14878760 to 33530993)	72.8	4672370 (3045241 to 6862799)	14.9	3857057 (2513856 to 5665264)	12.3

Saudi Arabia	214011283 (136788496 to 319642338)	69.2	44224875 (28266987 to 66053258)	14.3	51028702 (32615754 to 76215297)	16.5
Sudan	5216250 (3215566 to 8181564)	22.7	2297907 (1416549 to 3604213)	10.0	15487896 (9547541 to 24292397)	67.4
Syrian Arab Republic	13503528 (8339099 to 21073392)	45.3	298091 (184086 to 465196)	1.0	16007493 (9885422 to 24981040)	53.7
Tunisia	14615584 (9087987 to 22946415)	57.1	1279823 (795796 to 2009318)	5.0	9701062 (6032131 to 15230633)	37.9
United Arab Emirates	58910485 (38076231 to 88427266)	52.3	39649122 (25626832 to 59515101)	35.2	14079944 (9100438 to 21134624)	12.5
Yemen	607917 (377227 to 941802)	10.2	530437 (329149 to 821769)	8.9	4827576 (2995623 to 7479018)	81.0
Europe	8598825754 (5640398850 to 12634348211)	72.0	1031178256 (675789937 to 1513892428)	8.6	2322055976 (1516043610 to 3427146964)	19.4
Albania	9200474 (5901796 to 13783348)	54.0	238531 (153010 to 357346)	1.4	7598910 (4874446 to 11384025)	44.6
Andorra	713707 (449660 to 1074302)	69.5	178683 (112577 to 268962)	17.4	134526 (84756 to 202494)	13.1
Armenia	2942086 (1863467 to 4430229)	12.4	664342 (420783 to 1000374)	2.8	20120075 (12743709 to 30297048)	84.8
Austria	146580896 (94197051 to 218719150)	73.0	15862864 (10193927 to 23669607)	7.9	38351988 (24646078 to 57226517)	19.1
Azerbaijan	7704919 (4877086 to 11696879)	31.7	72917 (46155 to 110696)	0.3	16527901 (10461888 to 25091096)	68.0
Belarus	31017386 (19940308 to 46606448)	70.4	1718293 (1104648 to 2581891)	3.9	11323108 (7279345 to 17014002)	25.7
Belgium	158778646 (102078183 to 236353376)	76.8	10543894 (6778629 to 15695341)	5.1	37627231 (24190403 to 56010826)	18.2
Bosnia and Herzegovi-na	12799160 (8020300 to 19764823)	68.7	353980 (221813 to 546625)	1.9	5477370 (3432268 to 8458309)	29.4
Bulgaria	36380025 (23599420 to 53738502)	59.2	1167602 (757414 to 1724715)	1.9	23966571 (15546915 to 35402054)	39.0

Croatia	33712722 (21402940 to 51141219)	81.5	2895571 (1838289 to 4392497)	7.0	4757010 (3020047 to 7216246)	11.5
Cyprus	5378368 (3510420 to 7880217)	56.0	1286967 (839993 to 1885623)	13.4	2938894 (1918194 to 4305976)	30.6
Czechia	131626756 (86283137 to 194387297)	81.5	7106230 (4658231 to 10494529)	4.4	22933742 (15033381 to 33868707)	14.2
Denmark	144542967 (93825238 to 213905181)	83.3	4338024 (2815883 to 6419723)	2.5	24639978 (15994218 to 36464029)	14.2
Estonia	9919821 (6508925 to 14646546)	74.4	199996 (131228 to 295293)	1.5	3199942 (2099653 to 4724692)	24.0
Finland	81256055 (53696529 to 118892614)	80.2	2431603 (1606879 to 3557884)	2.4	17629119 (11649870 to 25794657)	17.4
France	1029180798 (668415347 to 1519664438)	75.3	210483191 (136701147 to 310794586)	15.4	127109979 (82553290 to 187687640)	9.3
Georgia	7934794 (4898395 to 12372713)	40.8	2411555 (1488728 to 3760334)	12.4	9101675 (5618747 to 14192230)	46.8
Germany	1907210835 (1279110226 to 2756223667)	77.7	233185366 (156390568 to 336990024)	9.5	314186598 (210715713 to 454049716)	12.8
Greece	56224221 (35220785 to 86716414)	48.1	19520675 (12228422 to 30107362)	16.7	41145376 (25774878 to 63459829)	35.2
Hungary	75522899 (49558917 to 111541608)	68.0	4331460 (2842350 to 6397239)	3.9	31319791 (20552374 to 46256961)	28.2
Iceland	7598367 (5019057 to 11036676)	82.9	146651 (96870 to 213012)	1.6	1420684 (938424 to 2063552)	15.5
Ireland	91731493 (60577185 to 133842522)	74.6	16846132 (11124765 to 24579659)	13.7	14386843 (9500711 to 20991387)	11.7
Israel	55014594 (35807463 to 81146186)	64.8	12140566 (7901956 to 17907260)	14.3	17828804 (11604271 to 26297375)	21.0
Italy	624456978 (406358223 to 923865206)	73.9	23660075 (15396523 to 35004365)	2.8	196885624 (128121063 to 291286323)	23.3
Kazakhstan	31758733 (20107776 to 48217078)	59.9	3287214 (2081272 to 4990749)	6.2	17973640 (11379860 to 27288130)	33.9

Kyrgyzstan	3925655 (2462831 to 5961281)	51.4	183299 (114996 to 278348)	2.4	3528507 (2213673 to 5358195)	46.2
Latvia	9728549 (6309710 to 14396497)	60.6	610041 (395658 to 902751)	3.8	5731175 (3717106 to 8481104)	35.7
Lithuania	20930079 (13567422 to 31304374)	65.1	868068 (562704 to 1298338)	2.7	10384663 (6731609 to 15531971)	32.3
Luxembou-rg	10497331 (6777875 to 15580613)	85.9	549918 (355069 to 816214)	4.5	1173159 (757481 to 1741256)	9.6
Malta	5483490 (3633525 to 8010887)	63.1	191184 (126684 to 279302)	2.2	3006795 (1992392 to 4392657)	34.6
Monaco	394373 (253039 to 586008)	85.0	32942 (21136 to 48949)	7.1	37117 (23815 to 55154)	8.0
Montenegro	2750342 (1757104 to 4164369)	60.8	27142 (17340 to 41096)	0.6	1746106 (1115530 to 2643826)	38.6
Netherlands	248751995 (161067362 to 364452823)	65.9	88705188 (57436768 to 129964209)	23.5	40011702 (25907649 to 58622154)	10.6
North Macedonia	4756216 (3077825 to 7089367)	59.0	48368 (31300 to 72095)	0.6	3256799 (2107528 to 4854414)	40.4
Norway	124328181 (81500849 to 181201450)	85.8	434714 (284968 to 633572)	0.3	20141745 (13203518 to 29355480)	13.9
Poland	331593913 (217733354 to 489230970)	71.4	38082214 (25005791 to 56186190)	8.2	94741118 (62209530 to 139780277)	20.4
Portugal	120620673 (79829078 to 176262129)	60.9	17231525 (11404154 to 25180304)	8.7	60409368 (39980080 to 88275779)	30.5
Republic of Moldova	5703704 (3600127 to 8791460)	59.7	439481 (277397 to 677399)	4.6	3410758 (2152839 to 5257205)	35.7
Romania	197038513 (125556141 to 297761907)	80.1	2459907 (1567492 to 3717377)	1.0	46492233 (29625606 to 70258428)	18.9
Russian Federation	518580259 (337568818 to 765152209)	61.2	19489127 (12686410 to 28755720)	2.3	310131332 (201879391 to 457591027)	36.6
San Marino	527897 (332518 to 802927)	82.2	5138 (3236 to 7814)	0.8	109176 (68769 to 166055)	17.0
Serbia	38893961 (24651538 to 58965415)	58.4	3063565 (1941731 to 4644536)	4.6	24641722 (15618269 to 37358225)	37.0

Slovakia	41670881 (27046731 to 61527283)	78.8	1057637 (686465 to 1561606)	2.0	10153311 (6590066 to 14991419)	19.2
Slovenia	23211176 (14941119 to 34767647)	72.4	5129542 (3301905 to 7683458)	16.0	3750977 (2414518 to 5618529)	11.7
Spain	365336842 (235422368 to 548507586)	70.6	39328045 (25342918 to 59046142)	7.6	112809393 (72694159 to 169369198)	21.8
Sweden	204689424 (134596644 to 298802305)	84.9	2893137 (1902426 to 4223354)	1.2	33512167 (22036435 to 48920519)	13.9
Switzerland	120596025 (79149654 to 176020882)	32.1	160043323 (105039727 to 233597806)	42.6	95049204 (62382749 to 138732969)	25.3
Tajikistan	1749193 (1078634 to 2745265)	27.3	89702 (55315 to 140783)	1.4	4561997 (2813142 to 7159811)	71.2
Turkey	307778986 (195168484 to 466457129)	77.9	20544939 (13027935 to 31137061)	5.2	66771051 (42340788 to 101195449)	16.9
Turkmenis-tan	3438401 (2098748 to 5408173)	18.0	993316 (606305 to 1562361)	5.2	14670513 (8954656 to 23074872)	76.8
Ukraine	68628282 (44821234 to 101387806)	44.8	6280713 (4101943 to 9278795)	4.1	78279134 (51124220 to 115645466)	51.1
United Kingdom	1097147854 (732051314 to 1585111494)	79.5	46922047 (31307855 to 67790932)	3.4	235990293 (157460094 to 340948510)	17.1
Uzbekistan	20885856 (13116974 to 32251316)	41.6	401651 (252249 to 620218)	0.8	28969084 (18193495 to 44733196)	57.7
Southeast Asia	776110410 (500156683 to 1161483787)	43.8	257783155 (166123202 to 385932266)	14.5	738615681 (474567206 to 1111340224)	41.7
Bangladesh	12369474 (7750205 to 19074695)	18.6	5785722 (3625096 to 8922035)	8.7	48347352 (30292470 to 74555396)	72.7
Bhutan	930043 (600820 to 1380428)	73.6	109937 (71021 to 163176)	8.7	224929 (145307 to 333854)	17.8
India	243826192 (156254598 to 367474514)	32.8	92178195 (59071860 to 138923292)	12.4	407368150 (261059511 to 613951323)	54.8
Indonesia	244348173 (159964195 to 362945769)	48.9	81449391 (53321398 to 120981923)	16.3	173891952 (113839550 to 258292694)	34.8

Maldives	2443365 (1539350 to 3744608)	79.3	123247 (77647 to 188883)	4.0	508393 (320293 to 779143)	16.5
Myanmar	7813002 (4877953 to 12061728)	15.8	4104298 (2562469 to 6336224)	8.3	37581527 (23463571 to 58018438)	76.0
Nepal	9157037 (5935691 to 13446720)	24.8	6387772 (4140623 to 9380171)	17.3	21378727 (13857924 to 31393752)	57.9
Sri Lanka	21703397 (13938319 to 32818572)	47.2	3310688 (2126184 to 5006223)	7.2	20967689 (13465834 to 31706078)	45.6
Thailand	233048991 (149004189 to 347807970)	71.7	64031592 (40939784 to 95562301)	19.7	28277911 (18080006 to 42202641)	8.7
Timor-Leste	470735 (291364 to 728783)	55.9	302315 (187119 to 468038)	35.9	69052 (42740 to 106906)	8.2
Western Pacific	6208013280 (4003469712 to 9209714859)	66.3	702050506 (448270338 to 1050437279)	7.5	2456381558 (1566406722 to 3679103476)	26.2
Australia	346114386 (224010523 to 509702555)	71.7	59375271 (38428583 to 87438514)	12.3	77236125 (49988401 to 113741156)	16.0
Brunei Darussalam	1558606 (997258 to 2325353)	94.3	0 (0 to 0)	0.0	94211 (60280 to 140557)	5.7
Cambodia	5622730 (3678247 to 8294944)	24.3	2614685 (1710461 to 3857320)	11.3	14901391 (9748112 to 21983308)	64.4
China	2281426664 (1430536072 to 3459203899)	56.0	358509904 (224798526 to 543589184)	8.8	1434039617 (899194102 to 2174356737)	35.2
Cook Islands	67785 (43602 to 101407)	87.6	4643 (2986 to 6946)	6.0	4952 (3186 to 7409)	6.4
Fiji	602267 (388642 to 895024)	65.4	197072 (127170 to 292867)	21.4	121559 (78442 to 180647)	13.2
Japan	2731628506 (1800512453 to 3978401661)	83.9	104186069 (68672704 to 151738800)	3.2	420000092 (276836837 to 611697037)	12.9
Kiribati	52045 (32104 to 81592)	82.3	11130 (6865 to 17449)	17.6	63 (39 to 99)	0.1
Lao People's Democratic Republic	2914265 (1869366 to 4328562)	36.9	1674320 (1073999 to 2486870)	21.2	3301254 (2117602 to 4903358)	41.8
Malaysia	60545820 (38834129 to 91164998)	52.2	15310437 (9820125 to 23053218)	13.2	40131903 (25740630 to 60427374)	34.6
Marshall Islands	21363 (13301 to 32983)	41.2	24423 (15205 to 37706)	47.1	6067 (3777 to 9366)	11.7

Micronesia (the Federated States of)	10237 (6444 to 15601)	28.3	24888 (15667 to 37928)	68.8	1049 (660 to 1599)	2.9
Mongolia	3744000 (2334370 to 5762522)	56.6	568876 (354692 to 875578)	8.6	2301964 (1435267 to 3543035)	34.8
Nauru	29959 (19030 to 45671)	86.3	4339 (2756 to 6615)	12.5	382 (243 to 582)	1.1
New Zealand	65238182 (41971371 to 98110876)	75.6	10527855 (6773158 to 15832707)	12.2	10527855 (6773158 to 15832707)	12.2
Niue	4597 (2896 to 6973)	98.7	0 (0 to 0)	0.0	61 (38 to 92)	1.3
Palau	56622 (36075 to 85242)	45.0	54231 (34552 to 81643)	43.1	15099 (9620 to 22731)	12.0
Papua New Guinea	1662416 (1030043 to 2591985)	57.7	933488 (578395 to 1455465)	32.4	285233 (176732 to 444725)	9.9
Philippines	61820610 (40993292 to 90038671)	40.6	16444891 (10904619 to 23951174)	10.8	74002011 (49070788 to 107780281)	48.6
Republic of Korea	507699300 (327170424 to 754081803)	59.5	87034166 (56086358 to 129271166)	10.2	257689393 (166059610 to 382744041)	30.2
Samoa	136708 (85392 to 211262)	72.6	32388 (20231 to 50051)	17.2	19019 (11880 to 29391)	10.1
Singapore	32984356 (21007115 to 49370984)	50.2	12878354 (8201981 to 19276320)	19.6	19843179 (12637747 to 29701269)	30.2
Solomon Islands	189165 (119235 to 291594)	74.7	55205 (34797 to 85097)	21.8	8610 (5427 to 13272)	3.4
Tonga	46954 (29306 to 72017)	58.1	25942 (16191 to 39789)	32.1	8001 (4994 to 12271)	9.9
Tuvalu	22096 (13906 to 33459)	74.3	7554 (4754 to 11438)	25.4	89 (56 to 135)	0.3
Vanuatu	90222 (58670 to 134129)	57.0	54449 (35408 to 80948)	34.4	13454 (8749 to 20002)	8.5
Viet Nam	103723419 (67676447 to 154329091)	43.8	31495924 (20550154 to 46862486)	13.3	101828927 (66440348 to 151510294)	43.0

Values in parentheses represent uncertainty intervals derived from the sensitivity analyses.

References

1. Chen N, Fong DYT, Wong JYH. Health and economic outcomes

associated with musculoskeletal disorders attributable to high body mass index in 192 countries and territories in 2019. JAMA Netw Open 2023 Jan;6(1):e2250674. https://doi.org/10.1001/jamanetworkopen.2022.50674.
2. The World Bank Group. Data bank world development indicators. Available from: https://databank.worldbank.org/source/world-development-indicators
3. World Health Organization (WHO). WHO global health expenditure database. Available from: https://apps.who.int/nha/database/Select/Indicators/en
4. International Labour Office Department of Statistics. Labour force by sex and age (thousands)-annual. Available from: https://www.ilo.org/shinyapps/bulkexplorer41/?lang=en&segment=indicator&id=EAP_TEAP_SEX_AGE_NB_A
5. United Nations Department of Economics and Social Affairs Population Dynamics. World population prospects. Available from: https://population.un.org/wpp/Download/Standard/Population/
6. International Labour Office Department of Statistics. Statistics on the working-age population and labour force. Available from: https://ilostat.ilo.org/topics/population-and-labour-force/#
7. Statistics Niue. Population. Available from: https://niue.prism.spc.int/category/population/
8. International Labour Office Department of Statistics. Statistics on labour income and inequality. Available from: https://ilostat.ilo.org/topics/labour-income/
9. International Labour Office Department of Statistics. Employment-to-population ratio by sex and age (%) annual. Available from: https://www.ilo.org/shinyapps/bulkexplorer2/?lang=en&segment=indicator&id=EMP_DWAP_SEX_AGE_RT_A
10. GBD 2019 Risk Factors Collaborators. Global burden of 87 risk factors in 204 countries and territories, 1990-2019: a systematic analysis for the Global Burden of Disease Study 2019. Lancet 2020 Oct;396(10258):1223-49. https://doi.org/10.1016/s0140-6736(20)30752-2.
11. Moher D, Liberati A, Tetzlaff J, Altman DG. Preferred reporting items for systematic reviews and meta-analyses: the PRISMA statement. PLoS Med 2009 Jul;6(7):e1000097. https://doi.org/10.1371/journal.pmed.1000097.
12. Dieleman JL, Cao J, Chapin A, Chen C, Li Z, Liu A et al. US health care spending by payer and health condition, 1996-2016. JAMA 2020 Mar;323(9):863-84. https://doi.org/10.1001/jama.2020.0734.
13. Institute for Health Metrics and Evaluation. GBD results tool. Available from: https://ghdx.healthdata.org/gbd-results-tool
14. The Economist Intelligence Unit. The global burden of cancer—challenges and opportunities. Available from: https://graphics.eiu.com/upload/eb/EIU_LIVESTRONG_Global_Cancer_Burden.pdf
15. Ding D, Lawson KD, Kolbe-Alexander TL, Finkelstein EA, Katzmarzyk PT, van Mechelen W et al. The economic burden of physical inactivity: a global analysis of major non-communicable diseases. Lancet 2016 Sep;388(10051):1311-24. https://doi.org/10.1016/s0140-6736(16)30383-x.

16. The World Economic Forum and the Harvard School of Public Health. The global economic burden of non-communicable diseases. Available from: https://www3.weforum.org/docs/WEF_Harvard_HE_GlobalEconomicBurdenNonCommunicableDiseases_2011.pdf
17. Dieleman JL, Baral R, Birger M, Bui AL, Bulchis A, Chapin A et al. US spending on personal health care and public health, 1996-2013. JAMA 2016 Dec;316(24):2627-46. https://doi.org/10.1001/jama.2016.16885.
18. Ebata-Kogure N, Murakami A, Nozawa K, Fujii K, Lin Y, Ushida T et al. Treatment and healthcare cost among patients with hip or knee osteoarthritis: a cross-sectional study using a real-world claims database in Japan between 2013 and 2019. Clin Drug Investig 2020 Nov;40(11):1071-84. https://doi.org/10.1007/s40261-020-00968-6.
19. Lee YR, Cho B, Jo MW, Ock M, Lee D, Lee D et al. Measuring the economic burden of disease and injury in Korea, 2015. J Korean Med Sci 2019 Mar;34(Suppl 1):e80. https://doi.org/10.3346/jkms.2019.34.e80.
20. Olafsson G, Jonsson E, Fritzell P, Hägg O, Borgström F. Cost of low back pain: results from a national register study in Sweden. Eur Spine J 2018 Nov;27(11):2875-81. https://doi.org/10.1007/s00586-018-5742-6.
21. Zhao X, Shah D, Gandhi K, Wei W, Dwibedi N, Webster L et al. Clinical, humanistic, and economic burden of osteoarthritis among noninstitutionalized adults in the United States. Osteoarthritis Cartilage 2019 Nov;27(11):1618-26. https://doi.org/10.1016/j.joca.2019.07.002.
22. Alonso-García M, Sarría-Santamera A. The economic and social burden of low back pain in Spain: a national assessment of the economic and social impact of low back pain in Spain. Spine (Phila Pa 1976) 2020 Aug;45(16):E1026-E32. https://doi.org/10.1097/BRS.0000000000003476.
23. Fisher S, Bellinger DC, Cropper ML, Kumar P, Binagwaho A, Koudenoukpo JB et al. Air pollution and development in Africa: impacts on health, the economy, and human capital. Lancet Planet Health 2021 Oct;5(10):e681-e8. https://doi.org/10.1016/s2542-5196(21)00201-1.
24. India State-Level Disease Burden Initiative Air Pollution Collaborators. Health and economic impact of air pollution in the states of India: the Global Burden of Disease Study 2019. Lancet Planet Health 2021 Jan;5(1):e25-e38. https://doi.org/10.1016/s2542-5196(20)30298-9.
25. Global Burden of Disease 2020 Health Financing Collaborator Network. Tracking development assistance for health and for COVID-19: a review of development assistance, government, out-of-pocket, and other private spending on health for 204 countries and territories, 1990-2050. Lancet 2021 Oct;398(10308):1317-43. https://doi.org/10.1016/s0140-6736(21)01258-7.
26. Kanal D, Kornegay JT. Accounting for household production in the national accounts: an update, 1965–2017. Available from: https://apps.bea.gov/scb/2019/06-june/0619-household-production.htm
27. Ahn YJ, Shin JS, Lee J, Lee YJ, Kim MR, Park KB et al. Evaluation of use and cost of medical care of common lumbar disorders in Korea: cross-sectional

study of Korean health insurance review and assessment service national patient sample data. BMJ Open 2016 Sep;6(9):e012432. https://doi.org/10.1136/bmjopen-2016-012432.
28. Flores NM, Nuevo J, Klein AB, Baumgartner S, Morlock R. The economic burden of uncontrolled gout: how controlling gout reduces cost. J Med Econ 2019 Jan;22(1):1-6. https://doi.org/10.1080/13696998.2018.1532904.
29. Williams EM, Walker RJ, Faith T, Egede LE. The impact of arthritis and joint pain on individual healthcare expenditures: findings from the medical expenditure panel survey (MEPS), 2011. Arthritis Res Ther 2017 Feb;19(1):38. https://doi.org/10.1186/s13075-017-1230-3.
30. Eriksson JK, Johansson K, Askling J, Neovius M. Costs for hospital care, drugs and lost work days in incident and prevalent rheumatoid arthritis: how large, and how are they distributed? Ann Rheum Dis 2015 Apr;74(4):648-54. https://doi.org/10.1136/annrheumdis-2013-204080.
31. Kalkan A, Hallert E, Bernfort L, Husberg M, Carlsson P. Costs of rheumatoid arthritis during the period 1990-2010: a register-based cost-of-illness study in Sweden. Rheumatology (Oxford) 2014 Jan;53(1):153-60. https://doi.org/10.1093/rheumatology/ket290.
32. Huscher D, Mittendorf T, von Hinüber U, Kötter I, Hoese G, Pfäfflin A et al. Evolution of cost structures in rheumatoid arthritis over the past decade. Ann Rheum Dis 2015 Apr;74(4):738-45. https://doi.org/10.1136/annrheumdis-2013-204311.
33. Kinge JM, Sælensminde K, Dieleman J, Vollset SE, Norheim OF. Economic losses and burden of disease by medical conditions in Norway. Health Policy 2017 Jun;121(6):691-8. https://doi.org/10.1016/j.healthpol.2017.03.020.
34. GBD 2019 Diseases and Injuries Collaborators. Global burden of 369 diseases and injuries in 204 countries and territories, 1990-2019: a systematic analysis for the Global Burden of Disease Study 2019. Lancet 2020 Oct;396(10258):1204-22. https://doi.org/10.1016/s0140-6736(20)30925-9.
